# The Protective Effect of *Chlorella vulgaris* Supplement Against Mercury Exposure Is Associated with DMT1: A Transcriptomic Analysis

**DOI:** 10.3390/plants15192896

**Published:** 2026-09-22

**Authors:** Chunmei Jin, Sabine M. Klauck, Xiao Lei, Dongxiao Li, Yuanping Hai, Thomas Efferth, Xiaohua Lu

**Affiliations:** 1 Department of Pharmaceutical Biology, Institute of Pharmaceutical and Biomedical Sciences, Johannes Gutenberg University Mainz, Staudinger Weg 5, 55128 Mainz, Germany; 2 Division of Cancer Genome Research, German Cancer Research Center (DKFZ), Im Neuenheimer Feld 280, 69120 Heidelberg, Germany; 3 National Center for Tumor Diseases (NCT) Heidelberg, Im Neuenheimer Feld 460, 69120 Heidelberg, Germany

**Keywords:** algal aqueous extract, heavy metal, DMT1, network pharmacology, mitotic catastrophe, ribosomal stress surveillance

## Abstract

Daily supplements have emerged as a compelling strategy for heavy metal detoxification (e.g., Hg and Pb), given that conventional chelation therapy lacks chronic exposure data and is heavily reliant on the doctor’s experience. Among such natural agents, *Chlorella vulgaris* (*C. vulgaris*), a commercially available supplement, is known for its notable heavy metal-binding capacity and ancillary benefits. Yet, the detoxification mechanisms of action of *C. vulgaris* have remained largely unexplored. In this study, our transcriptomic and signaling analyses demonstrated that the chemically characterized aqueous extract of *C. vulgaris* may have attenuated Hg^2+^-induced mitotic catastrophe in HEK-293 cells and was predicted to activate AKT signaling and modulate the expression of zinc finger protein-related genes. Furthermore, in corresponding DMT1-overexpressing cells, the protective effects of the extract were associated with the modulation of translation and ubiquitination pathways. These changes may coincide with the partial recovery of the two major ribosomal stress surveillance pathways. Intriguingly, under conditions in which Hg^2+^ did not provoke the more severe ribotoxic stress response, the *C. vulgaris* extract appeared to alleviate Hg^2+^-induced ribosomal stress by engaging both ribosomal quality control and the integrated stress response, particularly the response of GCN2 to amino acid deficiency. Ultimately, these findings suggest the protective effect of *C. vulgaris* extract against Hg^2+^ toxicity is associated with DMT1 at the transcriptome level, warranting further experimental investigation of its potential as a nutraceutical adjuvant in mercury detoxification.

## 1. Introduction

Despite the recognition of mercury toxicity, interest in its use has persisted across time and civilizations, resulting in pervasive human exposure. Mercury exposure comes from two principal forms: organic (mainly methylmercury, CH_3_Hg^+^) and inorganic mercury (including elemental mercury) [1]. Organic mercury exposure arises primarily from methylmercury in seafood consumption, and to a lesser extent, from the use of merbromin (C_20_H_8_Br_2_HgNa_2_O_6_) as an antiseptic and thimerosal (C_9_H_9_HgNaO_2_S, an ethylmercury-based compound), used as a preservative in children’s vaccines from the 1930s until its phase-out around 1999–2001 [2]. Inorganic mercury has historically been utilized in ancient medicine, skin-lightening products, topical antiseptics, diuretics, gold mining, and dental amalgam fillings [3]. However, organic mercury, primarily from fish consumption, has dominated concerns about human exposure, leaving the potential health risks of inorganic mercury underexplored. This study, therefore, addresses this gap by focusing on the toxicity of inorganic mercury, particularly, mercuric mercury (Hg^2+^).

Inorganic mercury has generally been regarded as having low neurotoxicity owing to poor absorption resulting from low lipophilicity and limited blood–brain barrier (BBB) penetration relative to methylmercury [3]. Mercuric ion (Hg^2+^) can nonetheless access the CNS by retrograde axonal transport from peripheral sites [4]. Of note, inorganic mercury is predominantly nephrotoxic, as Hg^2+^ preferentially accumulates in the kidney through reabsorption in the proximal tubule cells [5]. This retention is mediated by cysteine-conjugated complexes (Cys-S-Hg-S-Cys) that utilize basolateral organic anion transporters and luminal amino acid transporters [6]. Hg^2+^ toxicity stems from its high affinity for sulfhydryl groups (-SH) that form thiol-bound mercury with cysteine, glutathione, and numerous other proteins, thereby driving broad-spectrum toxicity through concurrent impairment of protein functions and the facilitation of molecular mimicry, enabling its cellular uptake through transporters [7]. Nonetheless, studies on the genotoxicity of mercuric chloride (HgCl_2_) have yielded conflicting results. It is generally accepted that inorganic mercury acts as a clastogen, causing chromosome breakage in eukaryotes [8]. Mechanistic data present a heterogeneous picture. Accumulated evidence suggests Hg^2+^ inhibits microtubule assembly by binding to thiol groups on tubulin, acting as a spindle inhibitor [8,9]. Conversely, another study attributed altered chromosome segregation not only to spindle protein binding but also to inactivation of RNA polymerase I [10]. Adding to this complexity, some studies indicated that HgCl_2_ failed to induce chromosome aberration in leukocytes [11], while others found it decreased the chromosome length, stimulated metallothionein synthesis, and induced chromatin fragmentation [12,13], underscoring the multifaceted nature of its genotoxic profile.

Chelation therapy is the primary treatment for heavy metal intoxication. In this context, 3-dimercapto-propanesulphonate (DMPS) is used for both inorganic and organic mercury, while dimercaptosuccinic acid (DMSA or succimer) is considered superior for organic mercury. However, both carry the risk of depleting essential elements and eliciting other adverse effects. Thus, nutritional interventions based on foods and supplements, which promote chelation and supply essential micronutrients, are emerging as a lifestyle support strategy for heavy metal detoxification [14,15]. Sulfur-rich foods such as garlic [16], broccoli [17], and the sulfur-containing amino acids taurine and methionine [18,19,20] are suggested for alleviating oxidative stress and facilitating detoxification of heavy metals. Alpha lipoic acid, a naturally occurring antioxidant in food that regenerates vitamins E and C, also possesses metal-chelating activity [14]. Cilantro was reported to enhance mercury excretion after dental amalgam removal and remains popular, though the evidence remains limited [21]. N-acetyl-cysteine (NAC), an orally available precursor of cysteine, can facilitate the function of a metal chelator [22]. Selenium, an essential trace mineral in *Chlorella* dietary supplements, forms stable complexes with mercury, acting as a potent antagonist to mercury toxicity. It even facilitates mercury transport through cell membranes by complex formation. A controlled trial among 103 mercury-exposed villagers demonstrated the benefit of selenium supplements [23]. Collectively, these lines of evidence point toward a prospect in which natural products may provide meaningful relief from mercury poisoning symptoms.

Living *Chlorella* cells possess a well-characterized metal-tolerance system, mainly through limiting entry, characterized by intracellular chelation by phytochelatins followed by vacuolar sequestration, active efflux, and antioxidant response [24]. However, processed *Chlorella* supplements instead use a passive physicochemical mechanism for detoxification, predominantly through the combined action of cell-wall metal-binding functional groups (e.g., carboxyl, amino, hydroxyl, phosphoryl), a rich nutrient profile, and antioxidant small molecules [25,26]. Several animal studies have demonstrated that *Chlorella* supplementation alleviates the heavy metal burden on the kidney and liver [27], increasing fecal and urinary excretion [25], for example, during methylmercury exposure [28], and additionally reducing intestinal absorption in some conditions [29]. For instance, it mitigates cadmium chloride-induced kidney dysfunction by reducing albumin and total protein loss in urine, while helping to restore normal renal tissue architecture [30]. Translation to humans nevertheless remains limited; no clinical recommendation for heavy metal detoxification has been established. A meta-analysis concluded that *Chlorella* supplementation improved total cholesterol levels and low-density lipoprotein cholesterol levels, but it did not affect high-density lipoprotein cholesterol levels [25]. Further metadata to illuminate the direct mechanistic link to heavy metal detoxification modes of action of *Chlorella* supplementation is required to lay the groundwork for rigorous human studies.

Among the plethora of transporters governing metal homeostasis, few are as pivotal as DMT1 (Nramp2/DCT1), encoded by the *SLC11A2* gene. DMT1 is a proton-coupled symporter that mediates the transport of various divalent metals (e.g., Fe^2+^, Mn^2+^, Cd^2+^, Pb^2+^) and is driven by the inwardly directed H^+^ electrochemical gradient [31,32]. Interestingly, Hg^2+^ may also be transported by DMT1 through proton-coupled symport and ionic mimicry [33]. The cell-based studies supported the role of DMT1 in the intestinal uptake of Hg^2+^, quantified by a continuous-flow cold-vapour generation atomic fluorescence spectrometer (CV-AFS) [33,34], whereas conflicting research suggested that DMT1 does not mediate Hg^2+^ uptake in Xenopus oocytes assessed by electrophysiology and fluorescence [35], demanding further investigation of the relationship between DMT1 and Hg^2+^. Of particular relevance to this study, *Chlorella* exhibited efficacy comparable to ferrous sulfate in rats with iron deficiency anemia [36], hinting at a potential modulatory role of *Chlorella* on the DMT1 transporter. Thus, combined with the evidence that Hg^2+^ may have been linked preliminarily to DMT1, we investigated whether DMT1 contributes to HgCl_2_ toxicity and to the protective action of *Chlorella*.

The present work aims to test the involvement of DMT1 in both mercuric chloride toxicity and *Chlorella vulgaris*-mediated protection, and to characterize the transcriptional pathways engaged. To test this, a cellular model of *Chlorella*-mediated protection of Hg^2+^ was established initially via resazurin assay. Furthermore, leveraging transcriptome analysis, we found that the protective effect of *Chlorella* extract in Hg^2+^-induced toxicity was critically associated with modulation of translation and ubiquitination pathways via the DMT1 transporter and coexisted with recovery of the two main ribosomal stress surveillance pathways: ribosomal quality control and the integrated stress response. These transcriptome-based observations tentatively position *C. vulgaris* as a promising, multi-mechanism supplement for mitigating Hg^2+^ poisoning that merits further functional and in vivo studies.

## 2. Materials and Methods

### 2.1. Aqueous Extraction of Chlorella vulgaris

The aqueous extract of *C. vulgaris* was prepared according to the methodology described in our prior publication, which also characterized its secondary metabolite profile [37]. Briefly, five grams of the chemically characterized *C. vulgaris* powder (batch number PZN-16735896, Roquette Klötze GmbH & Co. KG, Klötze, Germany) were subjected to ultrasonication with 300 mL of distilled water. The resulting extract was filtered through Whatman filter paper and concentrated to dryness under vacuum at 40–50 °C using a rotary evaporator. The crude aqueous extract yield was 18.8% (mg extract per g dry biomass weight) and was preserved at –20 °C for subsequent UPLC-HRMS/MS analysis as well as biological activity assessments. Stock solutions were prepared at 20 mg/mL in distilled water. The aqueous extract of *C. vulgaris* is characterized by several common and valuable flavonoids: tetrahydroxymethoxyflavone, luteolin, and apigenin, which bear a flavone core; alongside methyl quercetin, which is in the flavonol structure.

### 2.2. Cell Culture

Human embryonic kidney 293 (HEK-293) cells and a derivative line stably overexpressing pIRES2 DsRed-Express2-DMT1 (isoform 1A-IRE(+)), referred to as HEK-293 DMT1 cells, were kindly provided by Prof. Matthias A. Hediger (University of Bern, Bern, Switzerland) [38]. HEK-293 cells were originally sourced from ATCC CRL-1573. *DMT1* codes for the divalent metal transporter 1, and its overexpression was verified with qRT-PCR in our lab [37]. Both cell lines were used for the experiments within 25 passages to minimize phenotypic drift and were maintained in DMEM (Invitrogen, Darmstadt, Germany) supplemented with 10% fetal bovine serum, 10 mM HEPES, 1 mM Na-pyruvate, and 100 μM minimal essential medium nonessential amino acids at 37 °C with 5% CO_2_. Additionally, to maintain DMT1 overexpression, 500 μg/mL geneticin (Sigma-Aldrich, Taufkirchen, Germany) was added to the culture medium of HEK-293 DMT1 cells.

### 2.3. Cytotoxicity Assay

The cytotoxicity of the aqueous extract of a commercial *C. vulgaris* supplement (Roquette Klötze GmbH & Co. KG, Klötze, Germany) was determined in our previous study, which confirmed non-cytotoxicity up to 100 µg/mL [37]. For the present work, cytotoxicity towards mercury(II) chloride (HgCl_2_, Sigma-Aldrich, Taufkirchen, Germany) was assessed using a resazurin (Promega, Mannheim, Germany) reduction assay, measuring conversion of resazurin to highly fluorescent resorufin by living cells. Cytotoxicity of HgCl_2_ was evaluated across a concentration range from 0.003 µM to 10 µM in 72 h. To assess the cytoprotective potential of the aqueous extract of *C. vulgaris*, cells exposed to 0.01 µM HgCl_2_ were simultaneously treated with 0.01 to 30 µg/mL *C. vulgaris* aqueous extract for 72 h. The concentration of 0.01 µM HgCl_2_ was chosen deliberately as a sub-cytotoxic dose. This sub-cytotoxic concentration leaves the cells alive, so the mechanism readout reflects a primary molecular response, rather than being confounded by widespread death and membrane collapse that dominate at cytotoxic doses, which would otherwise mask the transporter-specific signaling [39]. The concentration range of *C. vulgaris* aqueous extract was based on our previous paper, in which it was shown to be non-cytotoxic [37]. The fluorescence intensity was measured with Infinite M2000 Pro™ plate reader (Tecan, Crailsheim, Germany) at an excitation/emission wavelength of 544/590 nm. IC_50_ values were calculated from concentration–response curves using a four-parameter logistic model in GraphPad Prism 10 (GraphPad Software, Boston, MA, USA), and IC_50_ values are presented with profile-likelihood 95% confidence intervals (Table 1). The experiments were conducted in three independent biological replicates, with six replicates per experiment.

### 2.4. RNA Extraction

HEK-293 and HEK-293 DMT1 cells were seeded at 1 × 10^6^ cells/well in a 6-well plate overnight. Afterwards, cells were either left untreated (negative controls), treated with HgCl_2_ alone (0.01 µM), or co-treated with HgCl_2_ (0.01 µM) and *C. vulgaris* aqueous extract (30 µg/mL) for 24 h, with *n* = 2 independent biological replicates per group. The concentrations of HgCl_2_ and *C. vulgaris* aqueous extract were selected according to the optimal restoration of cell viability in Hg^2+^-exposed HEK-293 DMT1 cells following treatment with *C. vulgaris* aqueous extract. Total mRNA was isolated using the InviTrap^®^ Spin Universal RNA Mini Kit (Invitek Molecular, Berlin, Germany) according to the manufacturer’s instructions. Afterwards, the total RNA concentrations and purity were determined using the NanoDrop1000 Spectrophotometer (PEQLAB, Erlangen, Germany) by measuring A260/A280 ratios. RNA integrity was assessed using an Agilent Fragment Analyzer (Agilent Technologies, Santa Clara, CA, USA) with ProSize data analysis software (Version 5.0.1). All samples used for microarray and qRT-PCR analysis exhibited an RNA Quality Number (RQN) of 10.0 (scale 1–10), with 28S/18S ribosomal RNA ratios of 1.4–2.3 (mean 1.95), confirming high-quality, intact total RNA suitable for downstream transcriptomic analysis. The RNA samples were used for microarray analysis and qRT-PCR technical confirmation.

### 2.5. Microarray Hybridization

Total RNA from the control and treated samples underwent quality control, labeling, and hybridization to Affymetrix GeneChips^®^ with human Clariom™ S assays (Thermo Fisher Scientific, Waltham, MA, USA) at the Genomics and Proteomics Core Facility of the German Cancer Research Center (DKFZ, Heidelberg, Germany). The raw data were normalized using the robust multi-array average (RMA) method to correct for systematic differences between arrays. Normalized unscaled standard error (NUSE) was examined for each array for probe-level quality control. The detection-above-background (DABG) test was used to identify genes whose signal was significantly stronger than the array’s background noise. We considered a probe set detected in a sample if its DABG *p*-value was below 0.05, and excluded probe sets that were not detected from further analysis. Overall array quality was assessed with the arrayQualityMetrics package and standard oligo diagnostics. The per-sample intensity boxplot for confirming normalization, sample-to-sample Pearson correlation heatmap for assessing data quality, principal component analysis (PCA), and hierarchical clustering analysis were generated in R software (version 4.3.3), using base R *t*-test, readxl, pheatmap, and RColorBrewer packages (Supplementary Figure S1). Subsequent analysis was performed with the Chipster data analysis platform (CSC, Espoo, Finland) to identify candidate transcriptional changes. Genes were first pre-filtered by coefficient of variation (0.1) to remove low-variance genes. Differentially expressed genes (DEGs) were identified using the empirical Bayes moderated *t*-test and defined as |log_2_ Fold change| ≥ 0.6 [40,41], together with the Benjamini–Hochberg false discovery rate (FDR) adjusted threshold of *q* < 0.1. As the Benjamini–Hochberg procedure does not prescribe the threshold of the FDR level [42], the threshold must be chosen according to the tolerated proportion of false positives. Thus, we set it at 0.1 for exploring transcriptomic screens whose outputs were subsequently confirmed by qRT-PCR. Venn diagrams illustrating the overlapping genes were generated using an online tool at the website http://bioinformatics.psb.ugent.be/webtools/Venn/ (accessed on 16 March 2026).

### 2.6. Ingenuity Pathway Analysis (IPA)

To elucidate the biological impact of treatment, the curated differentially expressed genes (DEGs) were analyzed via Ingenuity Pathway Analysis (IPA) software (Version 01-23-01) (Ingenuity Systems, Redwood City, CA, USA) [43]. Pathway significance and predicted activation states in IPA were determined by two key metrics: *p*-value and activation Z-score. The enrichment *p*-value was derived from Fisher’s exact test to estimate the probability of chance overlap between the submitted DEGs list and a pre-defined pathway in the Ingenuity Knowledge Base, independent of regulation direction. A −log *p*-value > 1.3 was considered significant. Activation Z-score predicts the directional perturbation of the pathways, with Z > 0 indicating activation and Z < 0 indicating inhibition; pathways with |Z| > 2 were considered significant. This algorithm quantitatively evaluates the concordance between the observed gene expression changes within a pathway and the causal relationships in the Ingenuity Knowledge Base, thereby enabling the identification of functionally relevant signaling pathways modulated by the treatment conditions [44].

### 2.7. Quantitative Real-Time Reverse Transcription PCR (qRT-PCR)

To technically confirm the microarray results, two-step qRT-PCR was employed to examine the expression levels of eight genes of interest identified in the microarray, selected based on both high Log2 fold change and annotation to metal-handling or protein homeostasis-related genes, such as *TFR2*, *NMNAT2*, *ESCO2*, and *MESD*. We used aliquots of the same total RNA samples used for microarray analyses, specifically from the Hg^2+^ exposure and co-treatment of *C. vulgaris* water extract with Hg^2+^. Reverse transcription to cDNA was carried out with 1 µg of total RNA per sample using the LunaScript^®^ RT SuperMix Kit cDNA Synthesis Kit (New England Biolabs, Darmstadt, Germany) in accordance with the manufacturer’s instructions. Oligonucleotide primers specific to each target gene were designed on the NCBI Primer-BLAST website (https://www.ncbi.nlm.nih.gov/tools/primer-blast/, accessed on 27 March 2025) and subsequently synthesized by Eurofins Genomics (Ebersberg, Germany). The primers are listed in Table 2, with amplicon sizes ranging from 100 to 292 base pairs. Melting-curve analysis was performed for each reaction. A single peak per amplicon confirmed product specificity, without evidence of non-specific amplification. GAPDH served as an internal reference gene for normalization, as its Ct values remained stable across all experimental groups. No-template controls (NTCs) were included in every run and yielded no detectable amplification. The cycle quantification (Cq) values were calculated using CFX Manager Software (version 3.1). The relative gene expression was calculated using the comparative 2^−∆∆*C*T^ method, assuming approximately equal amplification efficiency between target and reference genes [45].

### 2.8. Statistics

Statistical analyses were performed in GraphPad Prism 10 (GraphPad Software, Boston, MA, USA). Data from the cell viability assay are presented as the mean ± standard deviation (SD) from three independent biological replicates. A four-parameter logistic model was used for calculating the IC_50_, 95% confidence interval, and R^2^ value. Two-way analysis of variance (ANOVA) was conducted, followed by Bonferroni’s post hoc test to adjust for multiple pairwise comparisons. Asterisks denote statistically significant differences as follows: * *p* < 0.05 and *** *p* < 0.001, while “ns” represents not significant.

## 3. Results

### 3.1. Protective Effect of Chlorella vulgaris on Hg^2+^-Exposed Cells Is Associated with DMT1

Mercuric chloride cytotoxicity associated with DMT1 was evaluated by comparing the sensitivity of wild-type HEK-293 cells and the corresponding DMT1-overexpressing cell line to HgCl_2_ treatment. Consistent with the established role of DMT1 in mercury uptake [33], HgCl_2_ showed high sensitivity towards the HEK-293 DMT1 cell line, with a 4.41-fold reduction of the IC_50_ value compared to wild-type cells, with an IC_50_ value of 3.84 μM (95% CI 2.43–6.09) towards HEK-293 cells and 0.87 μM (95% CI 0.75–1.00) towards HEK-293 DMT1 cells (Figure 1A, Table 1).

To further substantiate the cytoprotective role of *C. vulgaris* in Hg^2+^-induced toxicity, an in vitro model was established using its aqueous extract. The cell viability of 0.01 µM Hg^2+^-exposed cells and the co-treatment with *C. vulgaris* aqueous extract from 0.01 to 30 µg/mL of the HEK-293 and HEK-293 DMT1 cell lines was evaluated to identify optimal protective conditions mediated by the DMT1 transporter. The concentration of 0.01 µM HgCl_2_ maintained both cell lines with approximately 80% cell viability, placing this dose firmly in the sub-cytotoxic range defined by a reduction in viability less than 30% [39]. The aqueous extract of *C. vulgaris* was employed based on our prior study, which identified bioactive flavonoids (luteolin, apigenin) through secondary metabolic profiling, and established the selected concentration as non-cytotoxic [37]. In parental HEK-293 cells, the extract produced no significant protection (95% CI -3.4 to 11.0, *p* > 0.99, Figure 1B). Significant cell viability recovery was observed in DMT1-overexpressing cells after treatment with 10 µg/mL *C. vulgaris* water extract (95% CI 1.3 to 15.8, *p* < 0.05, Figure 1B). Treatment with 30 µg/mL *C. vulgaris* water extract showed maximum cell viability recovery with an increase of 12.28% from 83.92% to 96.20% (95% CI 5.1 to 19.5, *p* < 0.001, Figure 1B), suggesting a DMT1-associated enhancement of the protective effect.

### 3.2. Transcriptomic Responses of Hg^2+^Toxicity and Chlorella vulgaris Intervention

To investigate the potential mode of action of HgCl_2_ and the recovery mode of action of *C. vulgaris* extract on HEK-293 cells and HEK-293 DMT1 cells, transcriptomic profiling was conducted by mRNA microarray hybridization coupled with Ingenuity Pathway Analysis (IPA). The quality control of microarray data showed successful normalization, high within-cell-line correlation, and the absence of low-correlation arrays, indicating consistent data quality with no outlier samples. PCA and hierarchical clustering analysis revealed treatment-related patterns (Supplementary Figure S1). DEGs across all groups are listed in Supplementary Tables S1–S4. The results showed that HgCl_2_ exposure induced substantial transcriptional alterations, with 206 and 193 genes deregulated in HEK-293 cells and HEK-293 DMT1 cells, respectively. Co-treatment with *C. vulgaris* aqueous extract was associated with a smaller number of deregulated genes, with 153 and 79 in HEK-293 cells and HEK-293 DMT1 cells, respectively. Only 33 and 22 deregulated genes, respectively, were shared across treatment conditions in HEK-293 cells and HEK-293 DMT1 cells (Figure 2A).

To validate the microarray hybridization results, eight selected genes (*SLC5A10*, *MESD*, *ATG16L1*, *EGR1*, *ESCO2*, *NMNAT2*, *CCAR1*, and *TFR2*) were quantified using qRT-PCR in both cell lines. Total mRNA was isolated from HEK-293 cells and HEK-293 DMT1 cells following 24 h treatment with 0.01 µM HgCl_2_ alone or in combination with 30 µg/mL *C. vulgaris* aqueous extract. The concordance of the top eight upregulated and downregulated genes between microarray data and qRT-PCR was highly correlated, with the Pearson correlation coefficients (r) of 0.96 and 0.98 in HEK-293 and HEK-293 DMT1 cells, respectively, underscoring the reliability of the gene expression analysis (Figure 2B).

### 3.3. Chlorella vulgaris Alleviates Hg^2+^-Induced Mitotic Catastrophe in HEK-293 Cells

Furthermore, comprehensive transcriptomic analysis was conducted using IPA. The analysis in HEK-293 cells suggested multiple lines of evidence that *C. vulgaris* mitigated a transcriptomic signature consistent with mitotic catastrophe in Hg^2+^-exposed cells. First, *C. vulgaris* alleviated Hg^2+^-induced DNA damage, as shown in the comparative analysis of biofunctions and canonical pathways using IPA. Hg^2+^ exposure significantly enriched DNA damage-related pathways (−log *p*-value > 1.3), including the lesioning of DNA, excision repair, mismatch repair, cross-link repair of DNA, and DNA damage response of cells. These perturbations were markedly attenuated following *C. vulgaris* treatment, with all pathways reduced to non-significant levels (−log *p*-value < 1.3) (Figure 3A). In contrast, binding of double-stranded DNA and binding of DNA biofunctions were significantly enriched (−log *p*-value > 1.3), indicating a strong ameliorative effect. Consistently, Hg^2+^ exposure was predicted to inhibit the activation of DNA endogenous promoter biofunctions and double-stranded DNA break repair (Z < 0) biofunctions in HEK-293 cells. Notably, *C. vulgaris* treatment alleviated the observed suppression, as evidenced by the reversal of activation of DNA endogenous promoter (Z > 0), abrogation of the double-stranded DNA break repair inhibition prediction (Z-score = 0 or N/A), and the concurrent activation of binding of DNA biofunction (Z > 0). Together, these findings collectively indicate that *C. vulgaris* attenuated DNA damage-related transcriptomic signatures.

Then, we found predicted associations for the ameliorative effect of *C. vulgaris* on microtubule dynamics perturbation in Hg^2+^-exposed HEK-293 cells. Multiple microtubule-related biofunctions were significantly enriched in Hg^2+^-exposed HEK-293 cells (−log *p*-value > 1.3, Figure 3B), including quantity of astral microtubules, microtubule dynamics, growth of astral microtubules, destabilization of astral microtubules, length of astral microtubules, and instability of microtubules. Remarkably, all of these pathways were abrogated following *C. vulgaris* treatment (−log *p*-value < 1.3). Conversely, biofunctions enriched for annotations related to assembly of kinetochores, cycling of the centrosome, cross-linkage of microtubules, and delay in initiation of separation of centrosome functions emerged as new significant pathways after *C. vulgaris* treatment (−log *p*-value > 1.3). The biofunction of segregation of chromosome remained significant (−log *p*-value > 1.3) in both conditions, albeit with reduced significance upon *C. vulgaris* treatment. Importantly, an inhibition of microtubule dynamics was predicted in Hg^2+^-exposed HEK-293 (Z < 0), an effect that was abolished by *C. vulgaris* treatment (Z-score = 0 or N/A), demonstrating an attenuation of transcriptomic alterations associated with microtubule dynamics.

The characterization of mitotic catastrophe is the profound change in nuclear organization and morphology [46]. Consistent with the defining feature, our transcriptomic analysis predicted that in HEK-293 cells, Hg^2+^ exposure significantly disrupted nuclear biofunctions including organization of nucleoli, organization of the nucleus, and morphology of the nuclear envelope (−log *p*-value > 1.3), all of which were normalized by *C. vulgaris* treatment (−log *p*-value < 1.3, Figure 3C). Alongside this, the IPA results also predicted that Hg^2+^ induced a strong mitosis arrest in HEK-293 cells, with significant enrichment (−log *p*-value > 1.3) of the biofunctions G2/M phase, M phase, arrest in mitosis of kidney cell lines, and arrest in mitosis of epithelial cell lines, accompanied by the prediction of inhibited mitosis (Z < 0). Remarkably, *C. vulgaris* treatment significantly reversed the cell cycle arrest as indicated by predicted activation (Z > 0) of “Mitosis”, “Mitosis of epithelial cell lines”, and “Cell cycle progression”.

The comparative analysis of canonical pathways provided further evidence supporting the mitotic catastrophe prediction, especially within the cell cycle checkpoints pathway. IPA results showed that the cell cycle checkpoints pathway was significantly enriched (−log *p*-value > 1.3) in Hg^2+^-exposed HEK-293 cells and predicted its activation (Z > 0, Figure 3D). Both key metrics were reversed by *C. vulgaris* treatment (−log *p*-value < 1.3, Z < 0). A parallel effect of biofunctions analysis was observed in canonical pathway analysis for the DNA damage-related pathway “DNA double-strand break repair by non-homologous end joining”, which was also returned to baseline by *C. vulgaris* treatment (−log *p*-value < 1.3).

It should be noted that IPA biofunction categories are broad and partially overlapping annotations; enrichment of a given term therefore indicates statistical over-representation of associated genes within the Ingenuity Knowledge Base, rather than a direct measurement of biological activity. Altogether, the data suggested that DNA damage, microtubule dynamics, cell cycle checkpoint, and mitosis arrest pathways converged on nuclear morphological disruption, thereby driving a transcriptomic signature consistent with mitotic catastrophe in Hg^2+^-exposed HEK-293 cells. In contrast, *C. vulgaris* aqueous extract attenuated this response.

### 3.4. Mitotic Catastrophe-Related Gene Expression in HEK-293 Cells

We next sought to define a gene expression signature for *C. vulgaris*-mediated attenuation of Hg^2+^-induced mitotic catastrophe. We focused on the three pathways with opposing predicted activation states between Hg^2+^-exposed and *C. vulgaris* co-treated HEK-293 cells: activation of DNA endogenous promoter; mitosis; and cell cycle checkpoints. Within these pathways, eight genes exhibited significant inverse expression patterns; these were Histone deacetylase 10 (*HDAC10*), LDL receptor-related protein 6 (*LRP6*), Fas-associated death domain protein (*FADD*), Nuclear receptor subfamily 2 group C member 2 (*NR2C2*), Mediator complex subunit 21 (*MED21*), Kinetochore scaffold 1 (*KNL1*), Charged multivesicular body protein 2A (*CHMP2A*), and Nuclear receptor binding SET domain protein 2 (*NSD2*) (Figure 4). Of note, *HDAC10* and *KNL1* were both mapped in the mitosis pathway and also intersected with the activation of DNA endogenous promoter and cell cycle checkpoints pathways, respectively. Collectively, these eight genes may serve as a transcriptional signature of the *C. vulgaris*-alleviated Hg^2+^-induced mitotic catastrophe in HEK-293 cells.

### 3.5. Signaling Landscape Underlying Mitotic Catastrophe in HEK-293 Cells

To understand the broader context, we analyzed the signaling landscape surrounding mitotic catastrophe. Transcription-related pathways, including transcription of DNA, transcription of RNA, and activation of gene exhibited activated predictions (Z > 0) in Hg^2+^-exposed HEK-293 cells, collectively encompassing 41 out of 206 Hg^2+^-deregulated genes. Among these, six genes (*LRP6*, *FADD*, *NR2C2*, *NSD2*, *MED21*, and *HDAC10*) were implicated in the alleviation of mitotic catastrophe by *C. vulgaris* (Figure 5A). The metabolism of protein and metabolism of macromolecule pathways were inhibited (Z < 0), involving 79 of the 206 Hg^2+^-deregulated genes. Among these, *LRP6*, *FADD*, *CHMP2A*, *NR2C2*, *NSD2*, and *HDAC10* were associated with alleviation of mitotic catastrophe (Figure 5B). Of note, the generic transcription pathway was activated (Z > 0) in Hg^2+^-exposed HEK-293 cells. While *C. vulgaris* co-treatment attenuated this activation, with a pronounced impact on zinc finger protein-encoding genes (14 of the 18 genes), including the *NR2C2* and *HDAC10* genes (Figure 5C). In parallel, recruitment of protein pathways was inhibited (Z < 0) in Hg^2+^-exposed HEK-293 cells, implicating the *CHMP2A*, *FADD*, and *NSD2* genes (Figure 5D). Collectively, *LRP6*, *FADD*, *NR2C2*, *NSD2*, *MED21*, *CHMP2A*, and *HDAC10* emerge as candidate hubs bridging transcriptional activation and metabolic suppression during Hg^2+^-induced mitotic catastrophe in HEK-293 cells.

The fate of mitotic catastrophe includes apoptosis, autophagy, necroptosis, and other cell death modes [46]. Our transcriptomic analysis revealed the inhibition of autophagy in Hg^2+^-exposed HEK-293 cells, implicating the *CHMP2A*, *FADD*, and *MED21* genes (Figure 6A). Concurrently, cell survival was also inhibited in Hg^2+^-exposed HEK-293 cells, including the *FADD*, *KNL1*, and *NSD2* genes (Figure 6B). Moreover, PI3K/AKT signaling was inhibited and dysregulated by *LRP6* and *NSD2* genes (Figure 6C). Remarkably, *C. vulgaris* co-treatment reversed this pattern, conferring activation of both AKT and NF-κB with the joint modulation of *LRP6*, *NSD2*, and *FADD* (Figure 6D). Taken together, *C. vulgaris* aqueous may counteract Hg^2+^-induced mitotic catastrophe by restoring cell survival-related pathways, especially through activation of the AKT pathway.

### 3.6. Chlorella vulgaris Ameliorates Hg^2+^-Induced Translation and Ubiquitination Perturbations in DMT1-Overexpressing Cells

To delineate the detoxification mechanism of *C. vulgaris* in Hg^2+^ via DMT1, we compared the biofunctions and canonical pathways in Hg^2+^-exposed and *C. vulgaris* co-treated HEK-293 DMT1 cells. Hg^2+^ exposure significantly affected translation-related pathways, including initiation of translation of mRNA, initiation of translation of protein, translation, and translation of protein (Figure 7A). By contrast, *C. vulgaris* significantly affected nuclear export of mRNA biofunction (Figure 7A). Interestingly, *C. vulgaris* mitigated the inhibition of translation and translation of protein induced by Hg^2+^exposure in HEK-293 DMT1 cells (Figure 7B, Z < 0). Comparative canonical pathway analysis also offered detailed insight into protein translation-related pathways. *C. vulgaris* reversed the significance of the GAIT translation signaling pathway, response of EIF2K4 (*GCN2*) to amino acid deficiency, and processing of capped intron-containing pre-mRNA compared with Hg^2+^-exposed HEK-293 DMT1 cells (Figure 7C). Hg^2+^ exposure elicited a pronounced inhibition in the response of EIF2K4 (*GCN2*) to amino acid deficiency and nonsense-mediated decay pathways (Figure 7D, Z < 0), while a more modest inhibition appeared in the processing of capped intron-containing pre-mRNA pathway. Of note, *C. vulgaris* mitigated the inhibition of all three translation-related pathways in Hg^2+^-exposed HEK-293 DMT1 cells (Figure 7D, Z = 0 or N/A).

Following investigation of the translation pathway, we noted that *C. vulgaris* reversed the significance of ubiquitination-related pathways induced by Hg^2+^-exposure via the DMT1 transporter, including the protein ubiquitination pathway and E3 ubiquitin ligases ubiquitinate target proteins (Figure 7C). Notably, the protein ubiquitination pathway was also inhibited in Hg^2+^-exposed HEK-293 DMT1 cells (Z < 0), and *C. vulgaris* mitigated this pathway (Figure 7D).

SUMOylation frequently crosstalks with ubiquitination for the same lysine residues [47]. Our comparative biofunction analysis revealed that Hg^2+^ exposure significantly affected SUMOylation of protein biofunction, whereas *C. vulgaris* was associated with deSUMOylation of proteins (Figure 7A). Meanwhile, *C. vulgaris* co-treatment reversed the significance of SUMOylation of chromatin organization proteins, SUMOylation of DNA damage response and repair proteins, and SUMOylation of transcription cofactors compared with Hg^2+^-exposed HEK-293 DMT1 cells (Figure 7C). However, no direct predictions (Z = 0 or N/A) were observed for any SUMOylation pathways in either Hg^2+^-exposed or *C. vulgaris* co-treated HEK-293 DMT1 cells, limiting mechanistic inference.

### 3.7. Chlorella vulgaris Reversed Hg^2+^-Impaired Translation Initiation, Elongation, and Termination in DMT1-Overexpressing Cells

Comparative biofunctions and canonical pathways analysis underscore the capacity of *C. vulgaris* aqueous extract to restore translational homeostasis against DMT1-mediated Hg^2+^ toxicity. To gain a deeper mechanistic insight, we interrogated the three phases of “Eukaryotic translation: initiation, elongation, and termination”. Strikingly, a divergence pattern emerged between Hg^2+^-exposed (Figure 8) and *C. vulgaris* co-treated HEK-293 DMT1 cells across all three phases (Figure 9).

In the translation initiation process, Hg^2+^ attenuated the 60S ribosomal complex, the 80S ribosome, and the subunits of the eukaryotic translation initiation factor 2 (eIF2) heterotrimeric complex (EIF2S1, EIF2S2, EIF2S3) (Figure 8A). Additionally, Hg^2+^ significantly impaired the function of the 80S initiation complex by dysregulating the GTPase/eIF5B interaction during the correct positioning of ${Met\text{-}tRNA}_{i}$ (Figure 8A), which represents the final step bridging translation initiation and elongation [48]. *C. vulgaris* reversed nearly all Hg^2+^-induced impairments in the translation initiation process via the DMT1 transporter, with the eIF2 heterotrimeric complex remaining as an exception (Figure 9A).

In the eukaryotic protein elongation process, the 80S ribosome coordinates aminoacyl-tRNA and elongation factors (eEFs) as the key regulators [49]. Hg^2+^ exposure significantly impaired both the key intermediate 80S:aminoacyl-tRNA:mRNA:eEF1A:GTP and the post-translocation elongation state (80S ribosome:mRNA:peptidyl-tRNA with elongating peptide) (Figure 8B). Notably, *C. vulgaris* fully rescued both perturbations (Figure 9B).

In the translation termination process, GTP hydrolysis by eRF3 in the Me-Q185-ETF1 (methylated eRF1) dimer form drives the shift from a stable pre-termination complex (GTP-bound state) to the release-competent post-pretermination complex (GDP-bound state), coupling hydrolysis to efficient termination before elongation factors rebind [50]. Hg^2+^ exposure significantly impaired this interaction (Figure 8C), while *C. vulgaris* fully abrogated it (Figure 9C).

### 3.8. Chlorella vulgaris Reinstates Ubiquitination Homeostasis Following Hg^2+^-Induced Perturbation in DMT1-Overexpressing Cells

The clear and main protein ubiquitination pathway in Hg^2+^-exposed HEK-293 DMT1 cells prompted a deeper mechanistic interrogation (Z < 0). As shown in Figure 10A and 10B, Hg^2+^ exposure significantly downregulated the Ubiquitin-Conjugating Enzyme E2 A (*UBE2A*) gene, a crucial E2 enzyme in the initial stages of the ubiquitin-proteasome pathway. Downstream, proteasome PA700/20S, known as the 26S proteasome, was compromised, as evidenced by downregulation of its essential component PSMC1 (Proteasome 26S Subunit, ATPase 1), which is the motor for unfolding ubiquitinated substrates and gatekeeper for the 20S catalytic core [51]. This happened alongside Proteasome 26S Subunit, Non-ATPase 13 (*PSMD13*), which supports the overall assembly and structural integrity [52]. Hg^2+^ ions have a high affinity for the sulfhydryl group (-SH) in cysteine, forming stable Cys-S-Hg-S-Cys [53]. In accordance with this finding, the cysteine-type deubiquitinating enzyme UPS40 was significantly downregulated in Hg^2+^-exposed HEK-293 DMT1 cells, implicating that the DMT1 transporter may contribute to this affinity, thereby disrupting the ubiquitination dynamics. Interestingly, the BAG1/HSP70/STUB1 misfolded complex exhibited bidirectional regulation by J-domain proteins, including DNAJC14 and DNAJC9. Strikingly, the main ubiquitination pathway showed no gene regulation in *C. vulgaris* co-treated HEK-293 DMT1 cells, suggesting that *C. vulgaris* may restore it to the homeostatic state rather than inducing compensatory correction. Furthermore, as shown in Figure 10C, an inhibitory signature of macromolecule metabolism was observed, encompassing 71 of the 193 Hg^2+^-induced DEGs in the Hg^2+^-exposed HEK-293 DMT1 cells, whereas it was markedly diminished upon *Chlorella vulgaris* co-treatment.

### 3.9. Chlorella vulgaris Mitigates Hg^2+^-Impaired Ribosomal Stress-Surveillance Pathways in DMT1-Overexpressing Cells

Surprisingly, when we connected the translation- and ubiquitination-related pathway datasets, we identified that two of the three ribosomal stress-surveillance pathways (ribosome quality control, integrated stress response, and ribotoxic stress response) appeared as an inter-pathway node [54].

First, mitigation of the response of GCN2 to amino acid deficiency in *C. vulgaris* co-treated HEK-293 DMT1 cells (Z = 0 or N/A, Figure 11A) directly implicated the integrated stress response, as GCN2 kinase is one of the four main triggers [54,55], suggesting *C. vulgaris* aqueous extract may co-modulate the integrated stress response via GCN2 kinase. Furthermore, by comparing genes within pathways exhibiting the highest Z-score, we observed that *RPL36A* and *RPL41* genes-both of which are components of the 60S eukaryotic ribosomal subunit-were significantly reversed upon *C. vulgaris* co-treatment in the pathways of response of GCN2 to amino acid deficiency and nonsense-mediated decay (Figure 11A). Additionally, the proteasome-related genes *PSMC1* and *PSMD13*– were significantly downregulated in the protein ubiquitination pathway following Hg^2+^ exposure. This concurrent perturbation of the 60S ribosome and proteasome directed our attention to the ribosomal quality-control signaling pathway, in which the 60S ribosome ubiquitinates lysine residues on the nascent peptide for proteasome degradation. Strikingly, a divergent pattern before and after *C. vulgaris* co-treatment was observed, primarily driven by reversal of 60S ribosome (Figure 11B), implicating that *C. vulgaris* aqueous extract may help redirect the Hg^2+^-impaired ribosome quality control via the DMT1 transporter. Finally, overwhelmed ribosome collision will exceed the ribosome quality control capacity, inducing ribotoxic stress response [56]. However, we observed no alterations in the ribotoxic stress signaling pathway (Figure 11C), indicating that Hg^2+^ ions had not induced a substantial ribosome conformational change in our model.

Collectively, the ribosomal stress-surveillance pathways may act as a central hub linking Hg^2+^-induced translation and ubiquitination perturbations, as well as the protective effect of *C. vulgaris* aqueous extract via the DMT1 transporter.

### 3.10. RAR Activation Is Involved in the DMT1-Dependent Hg^2+^Ion Toxicity and Chlorella vulgaris Protective Effect

Evidence for disrupted retinoic acid receptor (RAR) activation, one of the key toxicity pathways related to the biologically active form of vitamin A [57], was identified in both Hg^2+^-exposed HEK-293 cells and HEK-293 DMT1 cells, with a more pronounced effect in DMT1-overexpressing cells (−log *p*-value > 1.3, Z = 2) compared with wild-type cells (−log *p*-value < 1.3, Z < 2, Figure 12A), suggesting that DMT1-mediated Hg^2+^uptake may exacerbate RAR activation pathway disturbances. Notably, no significant RAR activation signaling (−log *p*-value < 1.3) in *C. vulgaris* aqueous extract co-treated HEK-293 DMT1 cells was evident, underscoring its potential to restore RAR homeostasis. In addition, IPA-derived toxicity pathway analysis revealed that the cytochrome P450 panel and NRF2-mediated oxidative stress response were significantly affected (−log *p*-value > 1.3, Figure 12B) in Hg^2+^-exposed HEK-293 DMT1 cells, suggesting both xenobiotic metabolism and antioxidant defense pathways may contribute to the DMT1-mediated Hg^2+^ ion toxicity.

## 4. Discussion

Chelation therapy for heavy metals is highly reliant on the clinician’s experience in identifying chronic low-level metal exposure. However, often misleading results are obtained due to a lack of long-term reference data [58,59]. *Chlorella* supplementation holds promise due to its notable metal-binding capacity and broad nutritional profile, as well as its antioxidant properties. Yet, how *Chlorella* orchestrates mercury detoxification remains poorly understood. To address this gap, we established a mechanistic framework for *Chlorella*-mediated protection of mercury by targeting a critical endogenous protein for metal transport, DMT1, using combined genomic and functional cellular assays.

The first objective was to confirm the role of DMT1 in mercuric chloride toxicity. This was verified by the resazurin cell viability assay, in which mercuric chloride toxicity towards the DMT1-overexpressing cell line showed a 4.41-fold reduction in the IC_50_ value compared to wild-type cells, indicating that increased mercury sensitivity is consistent with DMT1 involvement, which aligns with other studies [33,34,60]. Furthermore, we aimed to construct a mechanistic model for assessing the protective effects of *C. vulgaris* aqueous extract against Hg^2+^ toxicity. The cell viability assay further established that *C. vulgaris* restored viability in Hg^2+^- exposed DMT1-overexpressing cells but produced no significant protection in wild-type cells, indicating a DMT1-associated cytoprotective effect. Among the concentrations tested, 30 µg/mL aqueous extract with 0.01 µM HgCl_2_ achieved the maximal recovery of cell viability in HEK-293 DMT1 cells. This concentration was therefore selected for further mechanistic study. This protective effect may be attributable to the antioxidant activity of the flavonoids luteolin and apigenin in the aqueous extract, which was demonstrated in our previous study [37], with luteolin additionally being able to bind metals through the oxygen atoms on its catechol and hydroxy groups [61,62].

We next proceeded with the transcriptomic analysis to map the cellular pathways dysregulated by Hg^2+^. A prominent mechanistic divergence between wild-type HEK-293 cells and HEK-293 DMT1 cells regarding Hg^2+^ toxicity was obvious, in which Hg^2+^ converged on the enrichment annotations related to mitotic catastrophe in HEK-293 cells, whereas HEK-293 DMT1 cells faced translational and post-translational dysfunction. Encouragingly, *C. vulgaris* aqueous extract proved effective in both cell lines, underscoring its broad protective effect while revealing DMT1-driven pathway specificity. In HEK-293 cells, Hg^2+^ toxicity was highly associated with mitotic catastrophe. Previous studies have shown that inorganic mercury causes cell cycle arrest in G0/G1, S phase, and G2/M arrest, depending on the concentration and cell type [63,64]. In our model, Hg^2+^ clearly enriched mitotic arrest-associated genes, consistent with earlier literature. The exogenous sources of mitotic catastrophe derive from altered DNA replication, cell cycle checkpoints, chromosome segregation, and/or microtubular dynamics, and are characterized by profound changes in nuclear organization and morphology [65]. Remarkably, our transcriptome results aligned perfectly with this signature. Although the extract did not strongly reverse the Hg^2+^-induced chromosome segregation, the significance of differential expression was reduced compared with Hg^2+^-exposed cells, concurrent with the significant alleviation of the severity of nuclear morphology, DNA damage, cell cycle arrest, and microtubular dynamics. Prior studies have documented the potential of inorganic mercury to induce mitotic catastrophe. For instance, the inhibition of mitogen-induced DNA synthesis by mercurials was mentioned in the 1990s [66], and an increase of 60% in nuclear damage was reported in a human fetal liver cell line following 0.5 µM HgCl_2_, escalating to 200% with a prolonged period of 7 days [7]. Our data provide the first transcriptome evidence that *Chlorella* mitigates the mercuric chloride-induced mitotic catastrophe.

Given the unique mitotic catastrophe signature observed, we therefore sought to dissect the comprehensive signaling underlying it. Intriguingly, we first identified an ameliorating effect reminiscent of our previous study on lead toxicity-specifically within the generic transcription pathway, once again dominated by the zinc finger proteins (ZFPs). This convergence highlights a broader detoxification role of ZFPs in *C. vulgaris*-mediated regulation of heavy metals. Zinc finger proteins rely on zinc ions for structural stability to bind targets, and toxic metals can displace these zinc ions [67,68]. Moreover, previous studies also link toxic metal exposure to metal-regulatory transcription factor-1 (MTF-1), a zinc finger protein that senses zinc status and coordinates metal homeostasis [69]. These results suggest that *Chlorella* may normalize mercury- and lead-induced transcriptional disruption through ZFP regulation in HEK-293 cells. Moreover, subsequent macromolecule metabolism, cell proliferation, and AKT signaling were restored by *C. vulgaris*, also indicating a systemic recovery.

Submicromolar HgCl_2_ has been shown to reversibly inhibit protein synthesis in astrocytes, and prolonged or higher concentrations lead to progressively slower reversibility [70]. Consistent with the translation-related perturbations observed here, our transcriptomic analysis revealed that Hg^2+^ inhibited the translation process in DMT1-overexpressing cells, indicating Hg^2+^ may impair the translation machinery in a DMT1-associated manner. Additionally, a recent study reported that growth factors in the *C. vulgaris* extract promoted cell growth in Chinese hamster ovary cells in both 2D and 3D cell culture by increasing protein expression [71]. This aligns with our observations linking protein function to *Chlorella* activity. Taken together, our findings suggest that *C. vulgaris* aqueous extract may modulate the translation pathways to recover Hg^2+^- impaired DMT1 function.

DMT1 is tightly regulated by the ubiquitin–proteasome system to prevent metal toxicity [72]. A Hg(II) coordination complex has been associated with the inhibition of the 26S proteasome activity in vitro [73]. These results support our finding that mercuric chloride inhibited the ubiquitination process in DMT1-overexpressing cells and suggest that Hg^2+^ may reduce DMT1 degradation via ubiquitination suppression, thereby facilitating more Hg^2+^ uptake. Our previous study measured the secondary metabolite profile of the *C. vulgaris* aqueous extract, which revealed two bioactive flavonoids (apigenin and luteolin), with apigenin being the most closely tied to ubiquitination regulation. Apigenin is a well-established controller of the ubiquitination pathway that specifically targets the chymotrypsin-like activity of the 26S proteasome [74], indicating that it may drive the recovery of ubiquitination. Luteolin’s role in ubiquitination has been investigated in fewer studies in normal cells than in cancer cells, where it promotes the ubiquitination of oncogenic proteins (e.g., STAT3 and AKT) [75,76]. One study on embryonic cardiomyocytes, however, found that luteolin potentially influenced ubiquitination through MDM2 (an E3 ubiquitin ligase) to protect H9C2 cells against apoptosis [77]. Altogether, *C. vulgaris* aqueous extract may detoxify Hg^2+^ through ubiquitination pathway modulation, with apigenin as a likely key contributor.

The study of ribosomes has undergone a renaissance over the past decade. Yet, it was unexpected to find that the detoxification mechanism of the microalga *C. vulgaris* intersects with this field. ZNF574 is a key component in the ribosomal quality control pathway to monitor ribosome assembly by targeting the 60S ribosome for ubiquitin–proteasome degradation in K562 lymphoblast cells. Furthermore, loss of ZNF574 leads to pre-60S ribosome accumulation dominated by eIF6, because release of eIF6 is the final step for 60S maturation. Moreover, ZNF574^−/−^ homozygous mutant zebrafish embryos display several morphological defects reminiscent of ribosomopathies [78]. In our study, we found *C. vulgaris* aqueous extract specifically reversed the 60S ribosome component RPL36A and RPL41 caused by Hg^2+^ and concurrently recovered the EIF2AK4 amino acid deficiency response, highlighting the targeted role of *C. vulgaris* aqueous extract in mitigating Hg^2+^ toxicity via the DMT1 transporter. Ribosome heterogeneity has been reported [79]. Likewise, the toxicity of mercury may also be mediated by cell membrane components [80]. EIF2AK4 (also called GCN2) is a key eIF2α kinase, which binds to the ribosome P-stalk on the 60S subunit that is critical for detecting stalled ribosomes during amino acid deprivation [81]. Notably, in our model, Hg^2+^ did not trigger ribotoxic stress signaling, a pathway in which ribosome collisions target ZAKα [82] suggesting that the recovery effect of *C. vulgaris* aqueous extract on Hg^2+^ toxicity via the DMT1 transporter may operate independently of ribotoxic stress signaling.

Finally, our transcriptome results revealed that retinoic acid receptor (RAR) activation may be involved in the DMT1-mediated inorganic mercury toxicity. However, a previous study found that at the mRNA level, neither rat pituitary gland cells nor male Wistar rats exhibited RAR induction in response to inorganic mercury [83]—a discrepancy that, as we discussed earlier, underscores the critical role of cell type in shaping the mercury toxicity profile.

As a key consideration, our model showed that an active concentration of the *C. vulgaris* aqueous extract was 30 µg/mL. While reaching this concentration in blood serum is improbable owing to the hepatic first-pass metabolism, this concentration is biologically achievable locally in the gut. Multiple in vitro and in vivo gut models have confirmed *Chlorella*’s interaction with the gut lining and contents [84,85,86]. Moreover, the DMT1 transporter is particularly prevalent in the duodenum [87], collectively suggesting the gut constitutes a potential experimental model for *Chlorella*-mediated Hg^2+^ detoxification.

## 5. Conclusions

This study provides evidence that *C. vulgaris* aqueous extract exerts a cytoprotective effect against Hg^2+^-induced toxicity in a DMT1-associated manner, with transcriptomic signatures suggesting a multi-layered protective response. Exploratory transcriptomic analysis indicated that in HEK-293 cells, the extract mitigated Hg^2+^-induced mitotic catastrophe, concurrently modulating the generic transcription pathway dominated by zinc finger proteins and upregulating transcripts of pro-survival AKT signaling. In DMT1-overexpressing cells, transcriptomic profiling suggested *C. vulgaris* aqueous extract counteracted Hg^2+^-induced inhibition of the translation and ubiquitination pathways and shifted expression of genes in the two main ribosomal stress surveillance pathways, which serve as a biological buffering mechanism to recover from DMT1-associated Hg^2+^ toxicity. Intriguingly, in our model, under conditions in which Hg^2+^ did not provoke a severe ribotoxic stress response, pathway analysis suggested *C. vulgaris* extract alleviated Hg^2+^-induced sub-ribotoxic levels of ribosomal stress by engaging both ribosomal quality control and the integrated stress response, particularly the response of GCN2 to amino acid deficiency. These predicted mechanisms provide a framework for future functional studies.

## Figures and Tables

**Figure 1:**
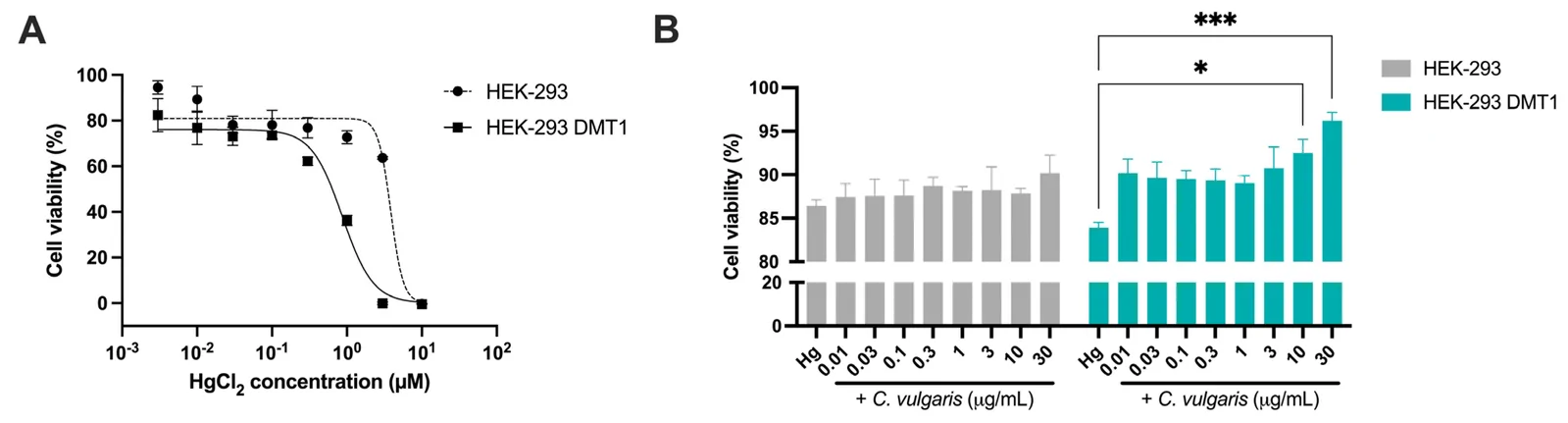
*Chlorella vulgaris* (*C. vulgaris*) aqueous extract exerts a cytoprotective effect against Hg^2+^-induced toxicity. (**A**) The dose–response curves of Hg^2+^ towards HEK-293 and HEK-293 DMT1 cell lines using a four-parameter logistic model. (**B**) Cell viability of 0.01 μM Hg^2+^-exposed and co-treatment from 0.01 to 30 μg/mL *C. vulgaris* aqueous extract in HEK-293 and HEK-293 DMT1 cell lines. All data are presented as mean ± SD from *n* = 3 independent experiments, with six replicates per experiment. Statistical significance was analyzed using two-way analysis of variance (ANOVA), followed by Bonferroni’s post hoc test comparing each co-treatment concentration with the Hg^2+^ alone control within each cell line, * *p* < 0.05, *** *p* < 0.001.

**Figure 2:**
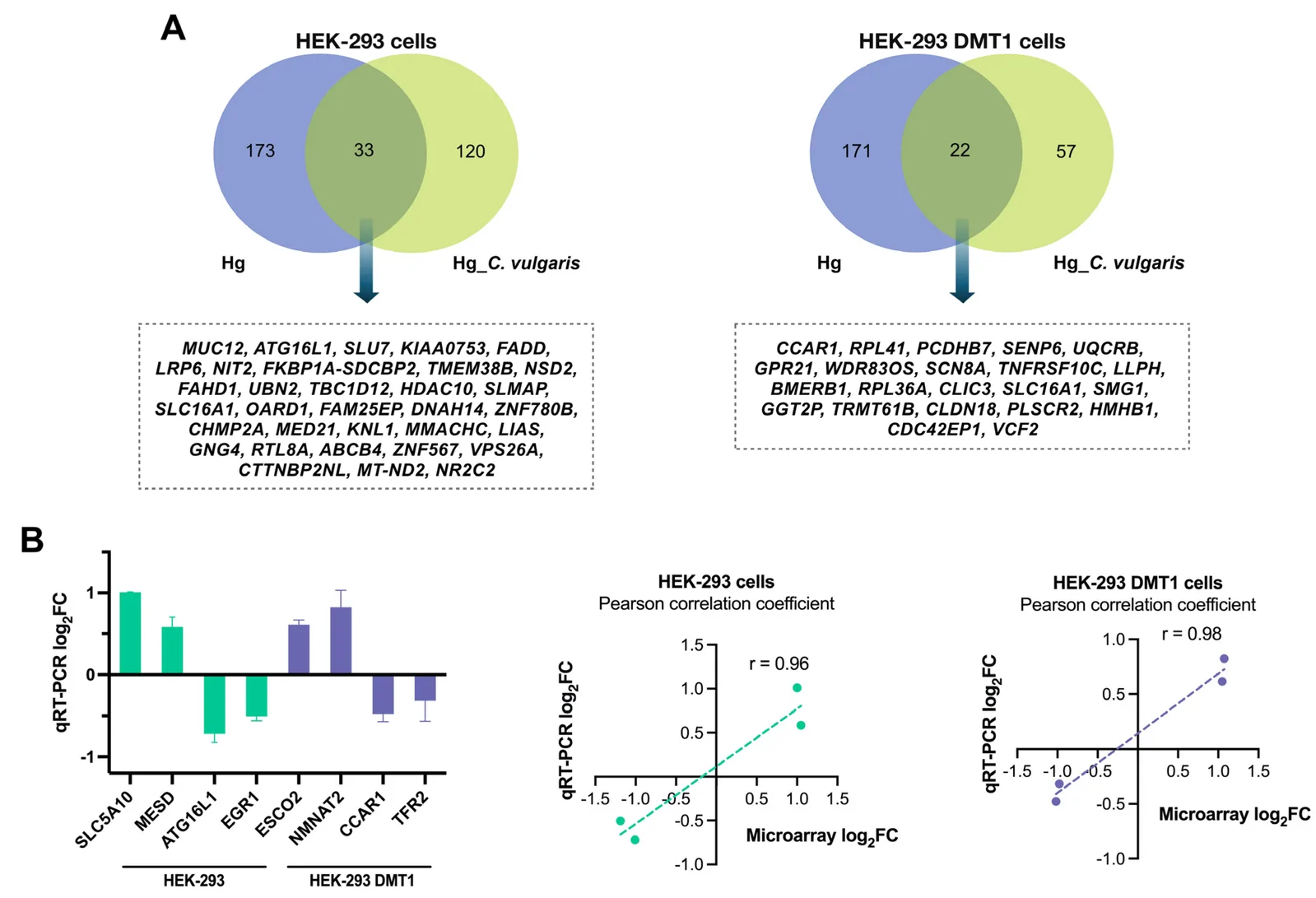
Preliminary analysis of microarray expression profiling. (**A**) Venn diagrams of deregulated gene expression upon treatment with 0.01 µM Hg^2+^ and co-treatment with 30 µg/mL *C. vulgaris* aqueous extract in HEK-293 cells and HEK-293 DMT1 cells. (**B**) Technical and biological verifications by qRT-PCR analyses in HEK-293 and HEK-293 DMT1 cells upon treatment with Hg^2+^ or Hg^2+^ plus *C. vulgaris*.

**Figure 3:**
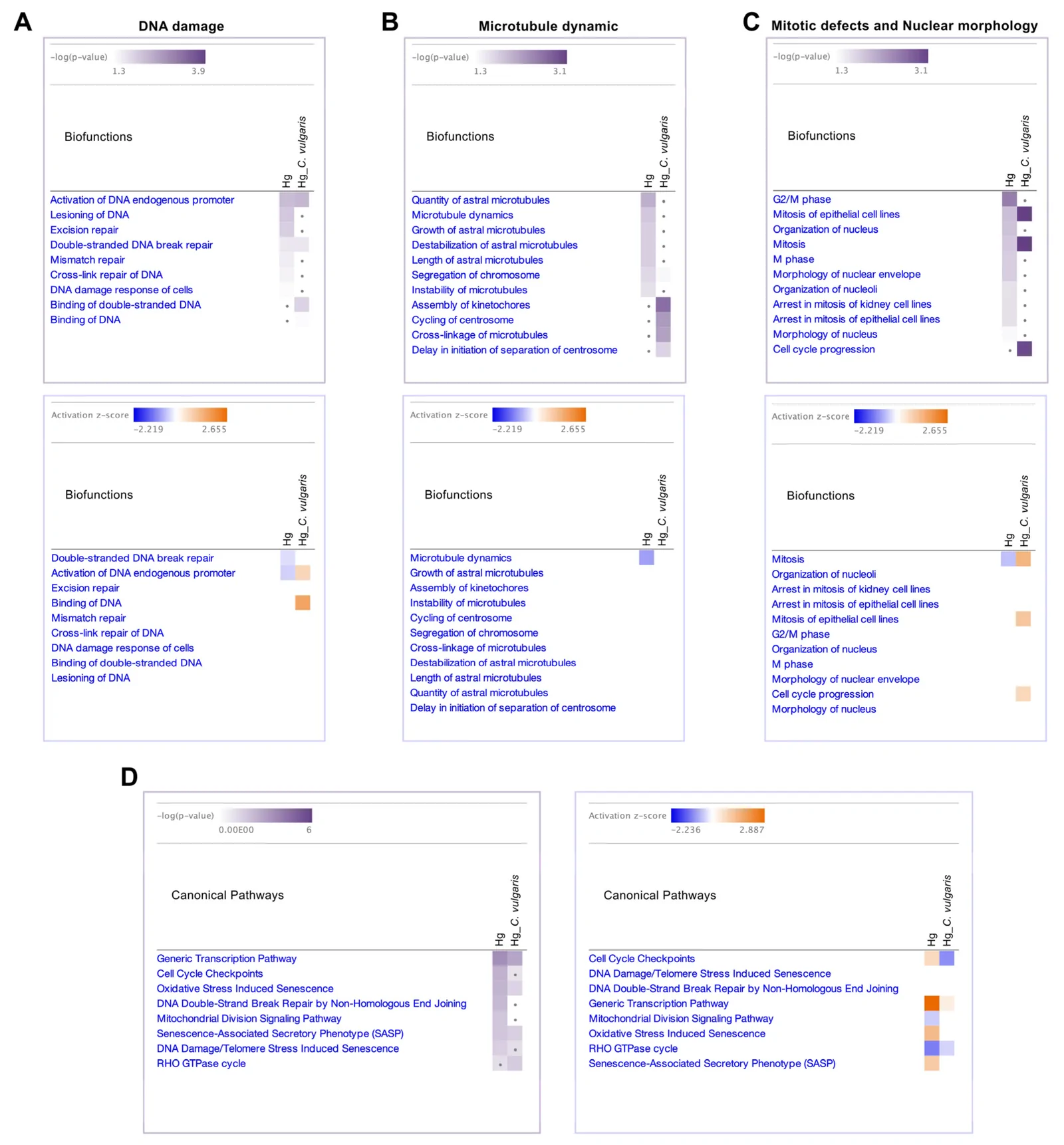
Comparative analysis of mitotic catastrophe-related biofunctions and canonical pathways among Hg^2+^-exposed and *C. vulgaris* co-treated HEK-293 cells. Biofunction heatmap of −log *p*-value and Z-score between Hg^2+^-exposed and *C. vulgaris* co-treated HEK-293 cells in (**A**) DNA damage-related biofunctions, (**B**) microtubule dynamics-related biofunctions, and (**C**) mitotic defects and nuclear morphology. (**D**) Canonical pathway heatmap of −log *p*-value and Z-score between Hg^2+^-exposed and *C. vulgaris* co-treated HEK-293 cells in mitotic defect-related pathways. Statistical significance was calculated based on the *p*-value and the Z-score. The color of the block indicates its predicted state: activation (orange), inhibition (blue). Darker colors indicate higher absolute *p*-values or Z-scores. The dot indicates no statistical significance.

**Figure 4:**
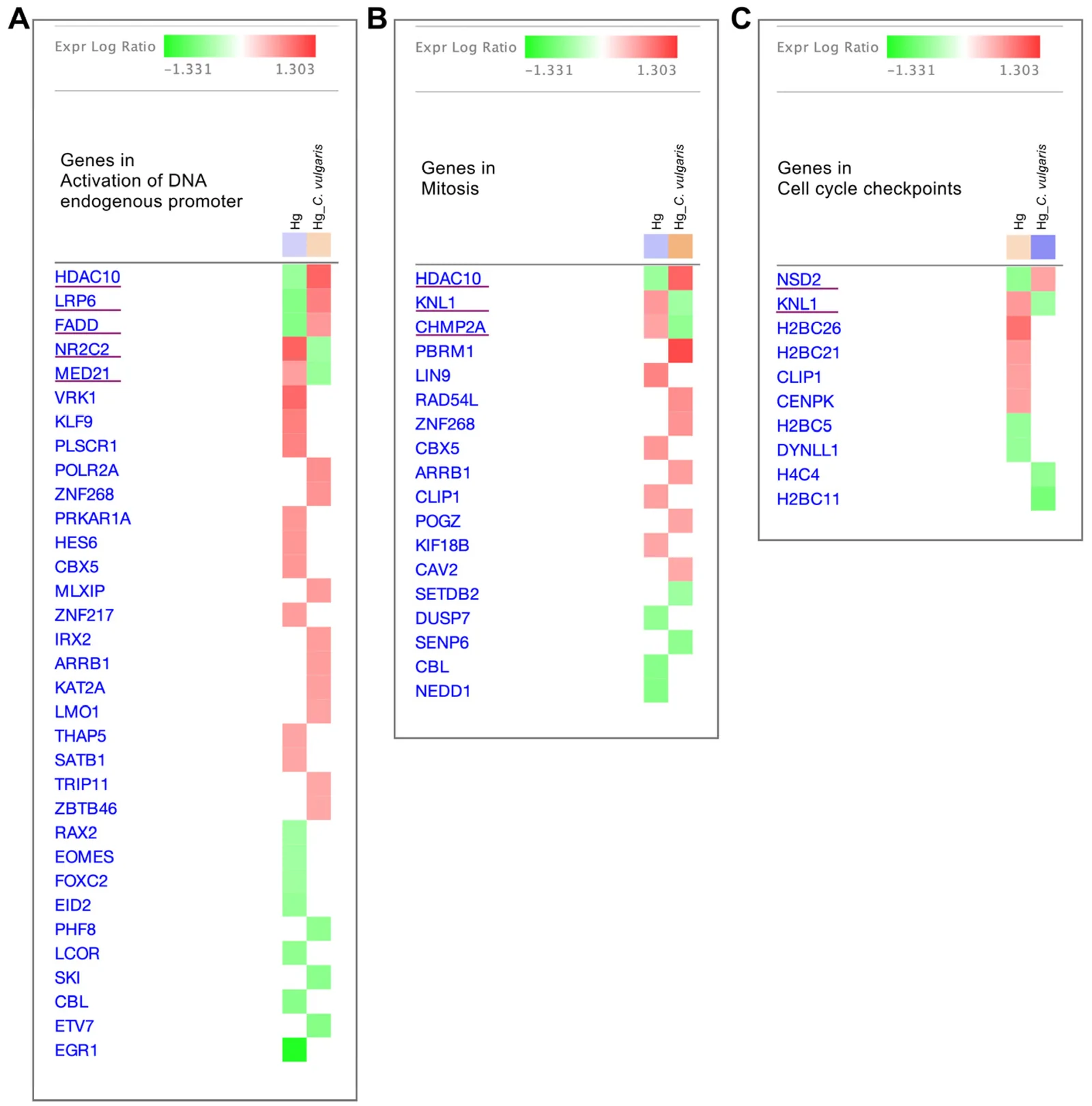
Gene expression heatmap of mitotic catastrophe-related pathways among Hg^2+^-exposed and *C. vulgaris* co-treated HEK-293 cells. (**A**) Activation of the DNA endogenous promoter, (**B**) Mitosis, (**C**) Cell cycle checkpoints. The color of the block indicates its expression direction: upregulated (red), downregulated (green). Darker colors indicate higher expression. The inversely expressed genes are underlined.

**Figure 5:**
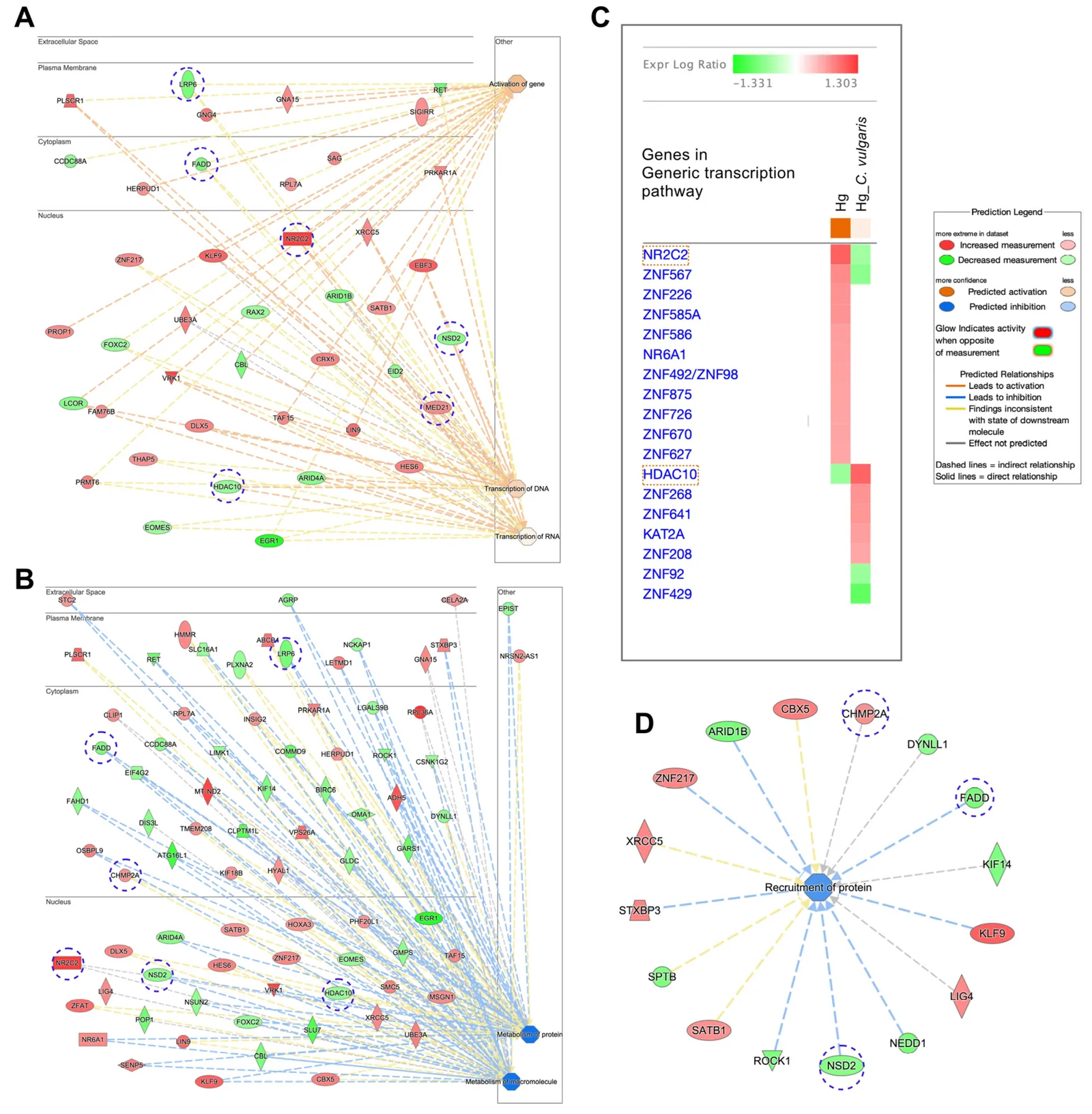
Mitotic catastrophe occurs concurrently with transcriptional activation and metabolic suppression in Hg^2+^-exposed HEK-293 cells. DEGs in (**A**) transcription of DNA or RNA and activation of gene pathways and (**B**) metabolism of protein and metabolism of macromolecule pathways in Hg^2+^-exposed HEK-293 cells. (**C**) Heatmap of genes in the generic transcription pathway among Hg^2+^-exposed and *C. vulgaris* co-treated HEK-293 cells. (**D**) Differentially expressed genes (DEGs) in the recruitment of protein pathways in Hg^2+^-exposed HEK-293 cells. The relevant genes are highlighted in dashed frames and dotted circles.

**Figure 6:**
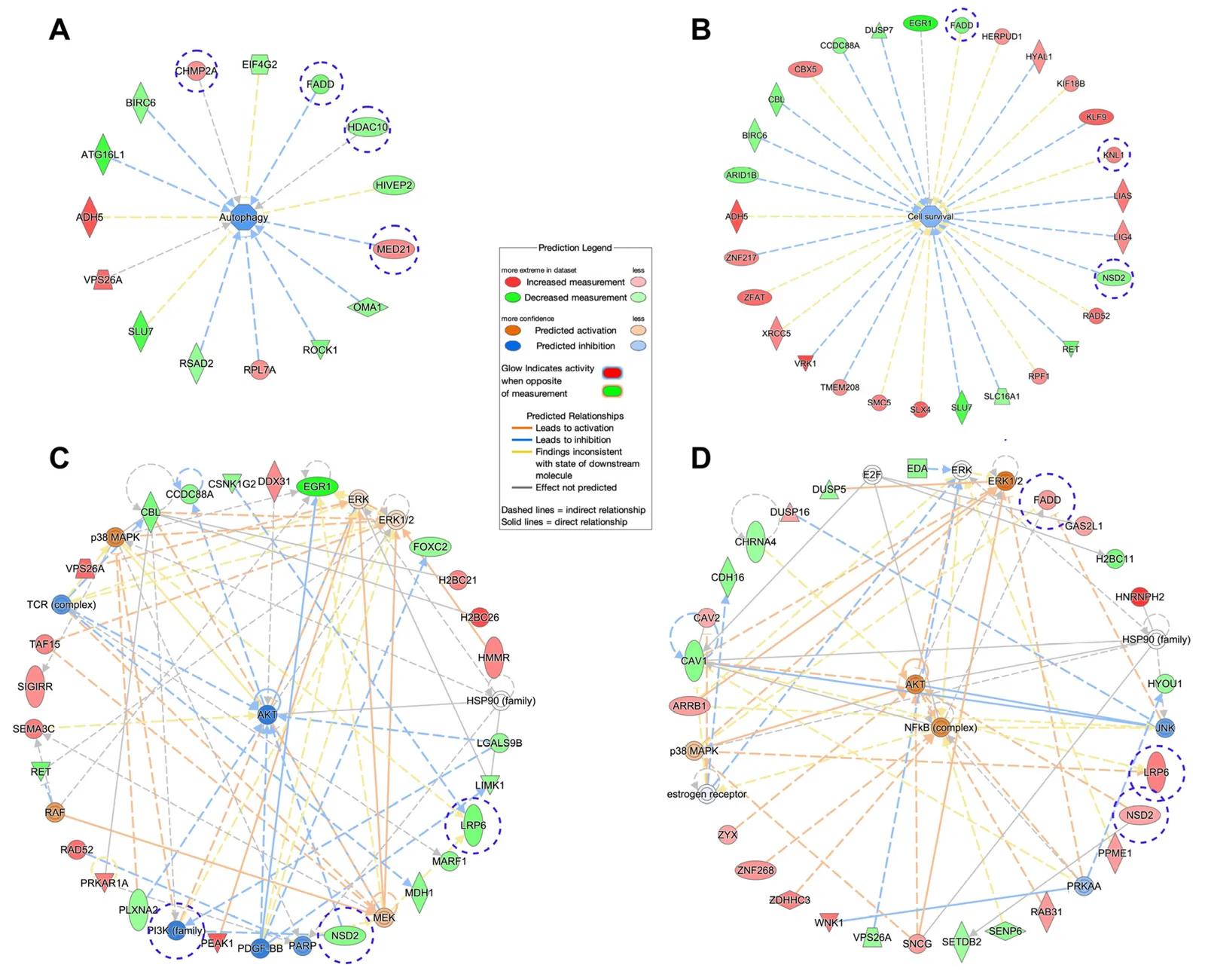
*C. vulgaris* aqueous extract counteracts Hg^2+^-induced mitotic catastrophe by restoring cell survival-related pathways in HEK-293 cells. DEGs of the (**A**) autophagy and (**B**) cell survival pathways in Hg^2+^-exposed HEK-293 cells. DEGs of the Akt network in (**C**) Hg^2+^-exposed HEK-293 cells and (**D**) *C. vulgaris* co-treated HEK-293 cells. The Z-score indicates the activation state, and the color indicates its direction: activation (orange), inhibition (blue). Darker colors indicate higher absolute Z-scores. Dashed circles and squares highlight genes associated with mitotic catastrophe.

**Figure 7:**
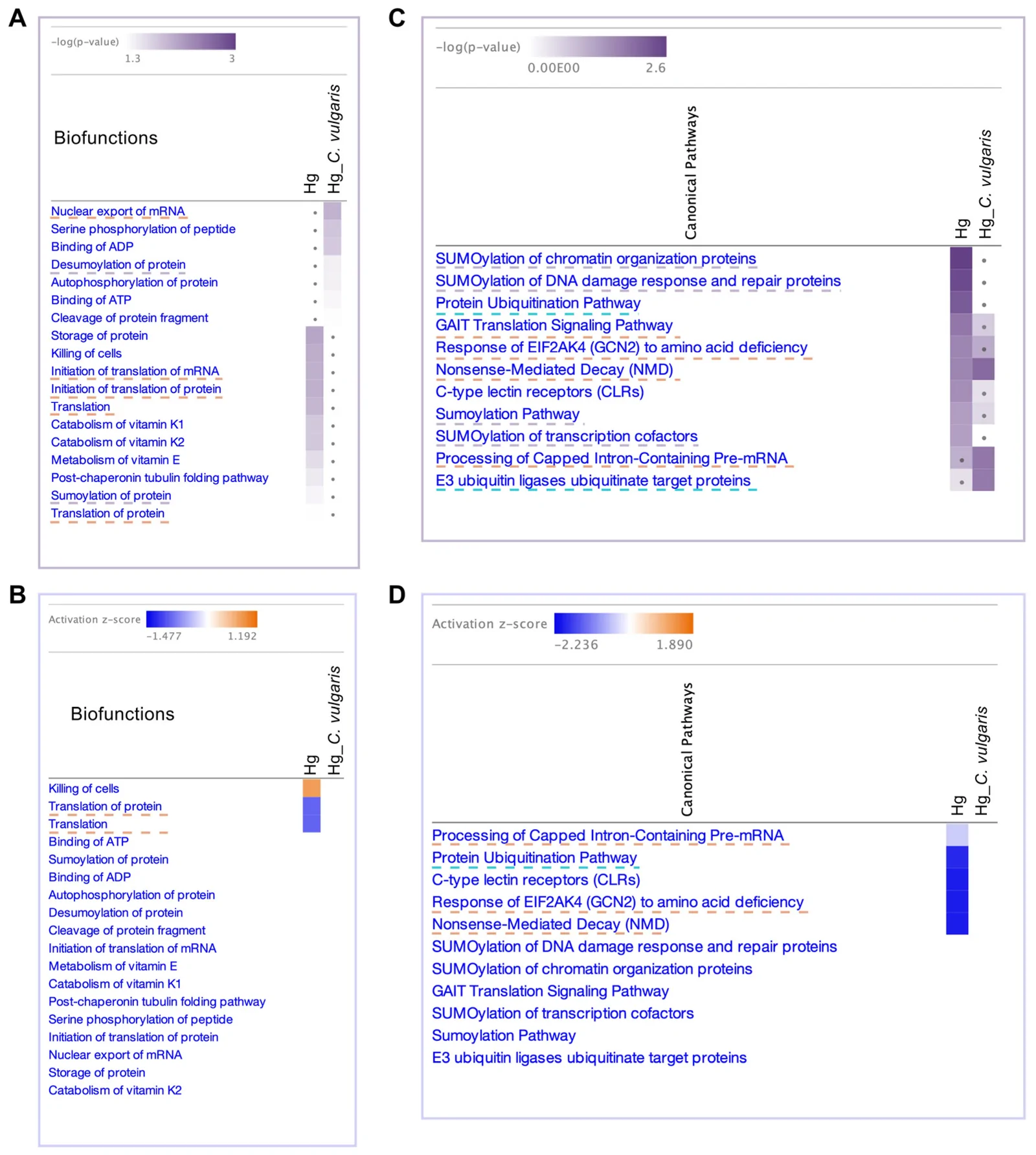
IPA-based comparative biofunctions and canonical pathways analysis depict a translation- and ubiquitination-related pattern in Hg^2+^-exposed versus *C. vulgaris* co-treated HEK-293 DMT1 cells. −log *p*-value and Z-score in the biofunction heatmap (**A**,**B**) and canonical pathways heatmap (**C**,**D**) between Hg^2+^-exposed and *C. vulgaris* co-treated HEK-293 DMT1 cells. Statistical significance was calculated based on the *p*-value and the Z-score. The color of the block indicates its predicted state: activation (orange), inhibition (blue). Darker colors indicate higher absolute *p*-values or Z-scores. The dot indicates no statistical significance. Pathways and Biofunctions related to translation, ubiquitination and SUMOylation are underlined with orange, green and purple, respectively.

**Figure 8:**
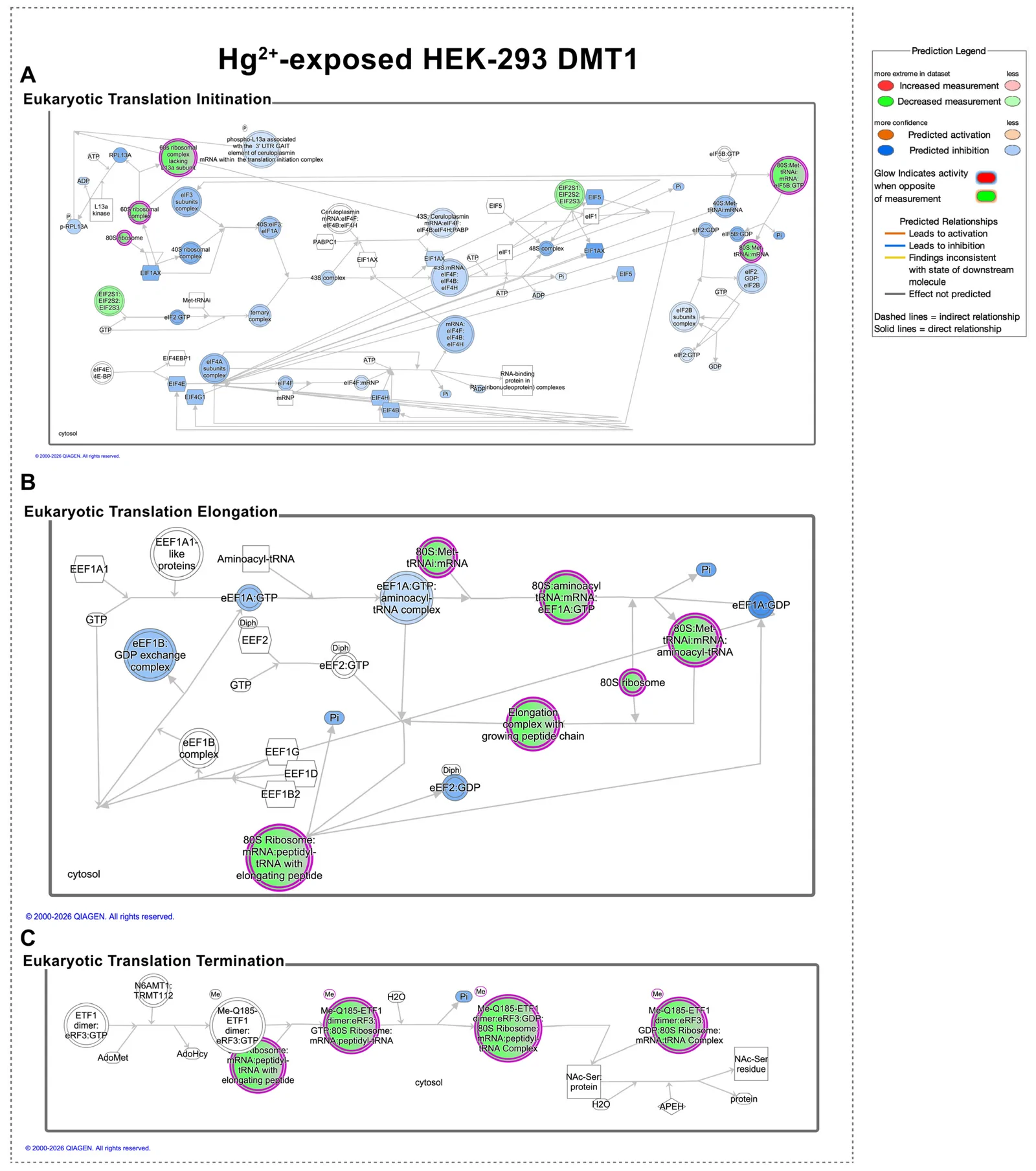
Three predicted phases of the eukaryotic translation process: (**A**) initiation, (**B**) elongation, and (**C**) termination in Hg^2+^-exposed HEK-293 DMT1 cells.

**Figure 9:**
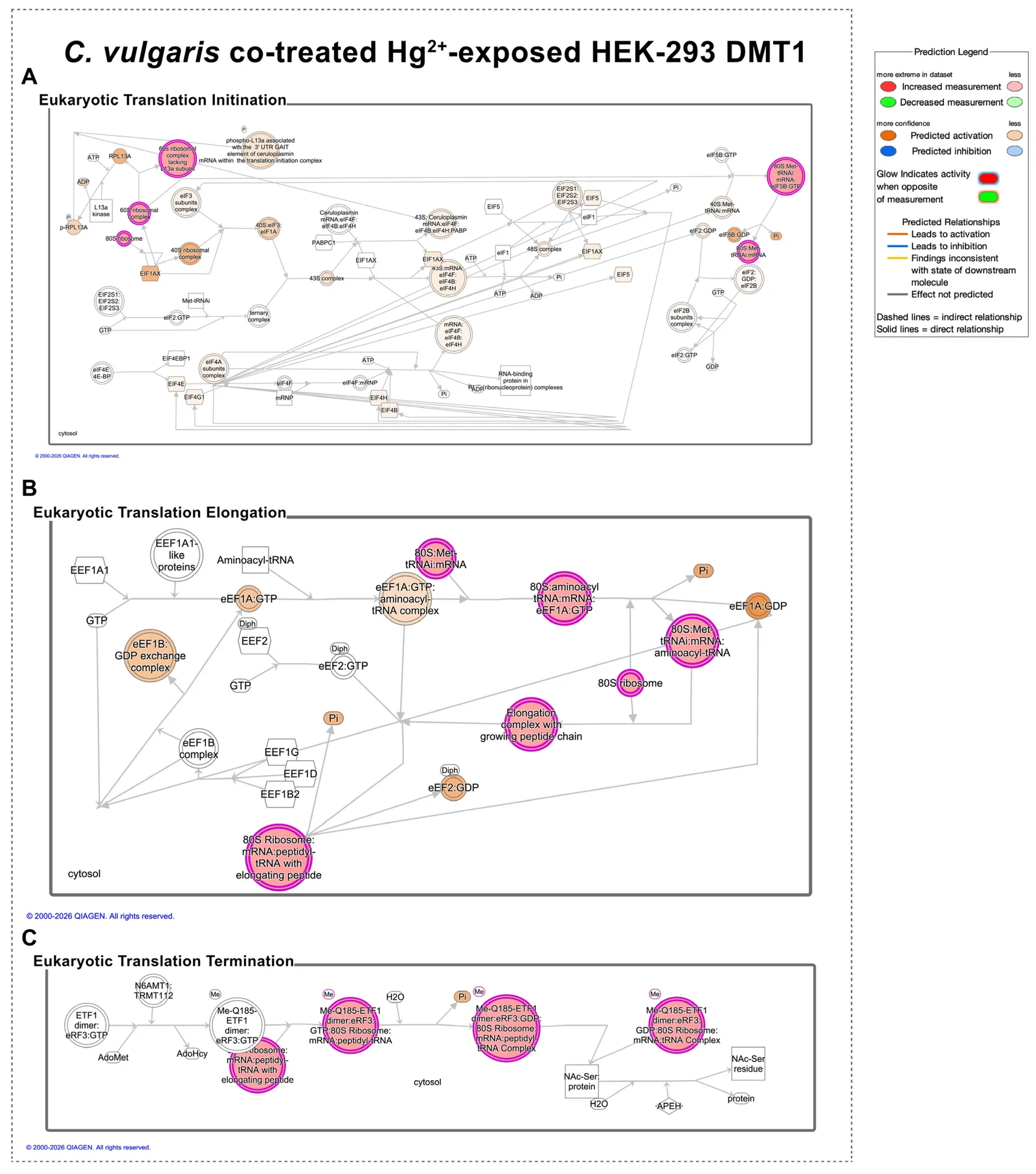
Three predicted phases of the eukaryotic translation process: (**A**) initiation, (**B**) elongation, and (**C**) termination in *C. vulgaris* co-treated Hg^2+^-exposed HEK-293 DMT1 cells.

**Figure 10:**
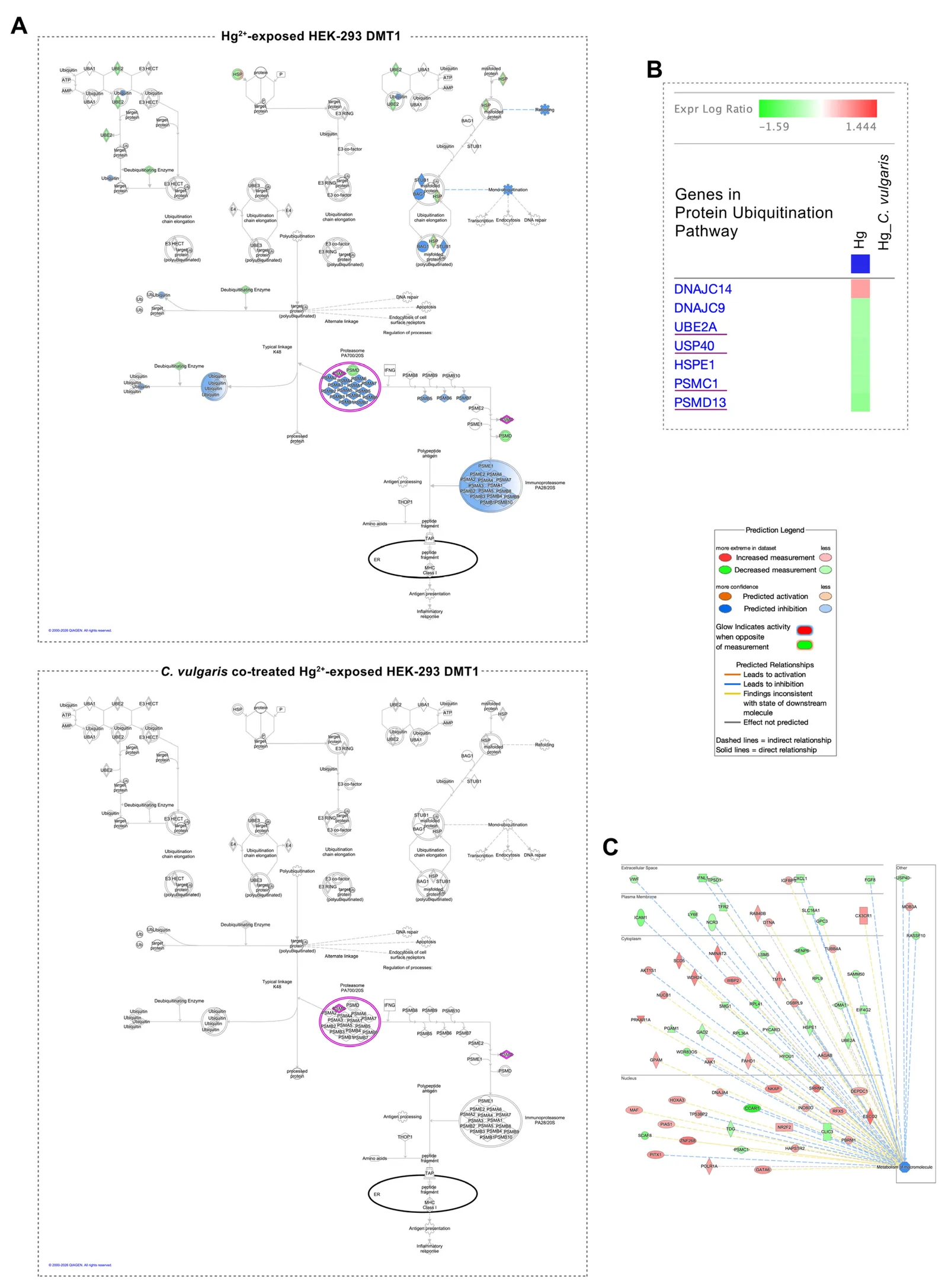
Ubiquitination signature in Hg^2+^-exposed and *C. vulgaris* co-treated HEK-293 DMT1 cells. (**A**) Protein ubiquitination pathway between Hg^2+^-exposed HEK-293 DMT1 cells and *C. vulgaris* co-treated HEK-293 DMT1 cells. (**B**) Gene expression heatmap of ubiquitination-related pathways among Hg^2+^-exposed and *C. vulgaris* co-treated HEK-293 cells. (**C**) Inhibition of the metabolism of macromolecule function in Hg^2+^-exposed HEK-293 DMT1 cells.

**Figure 11:**
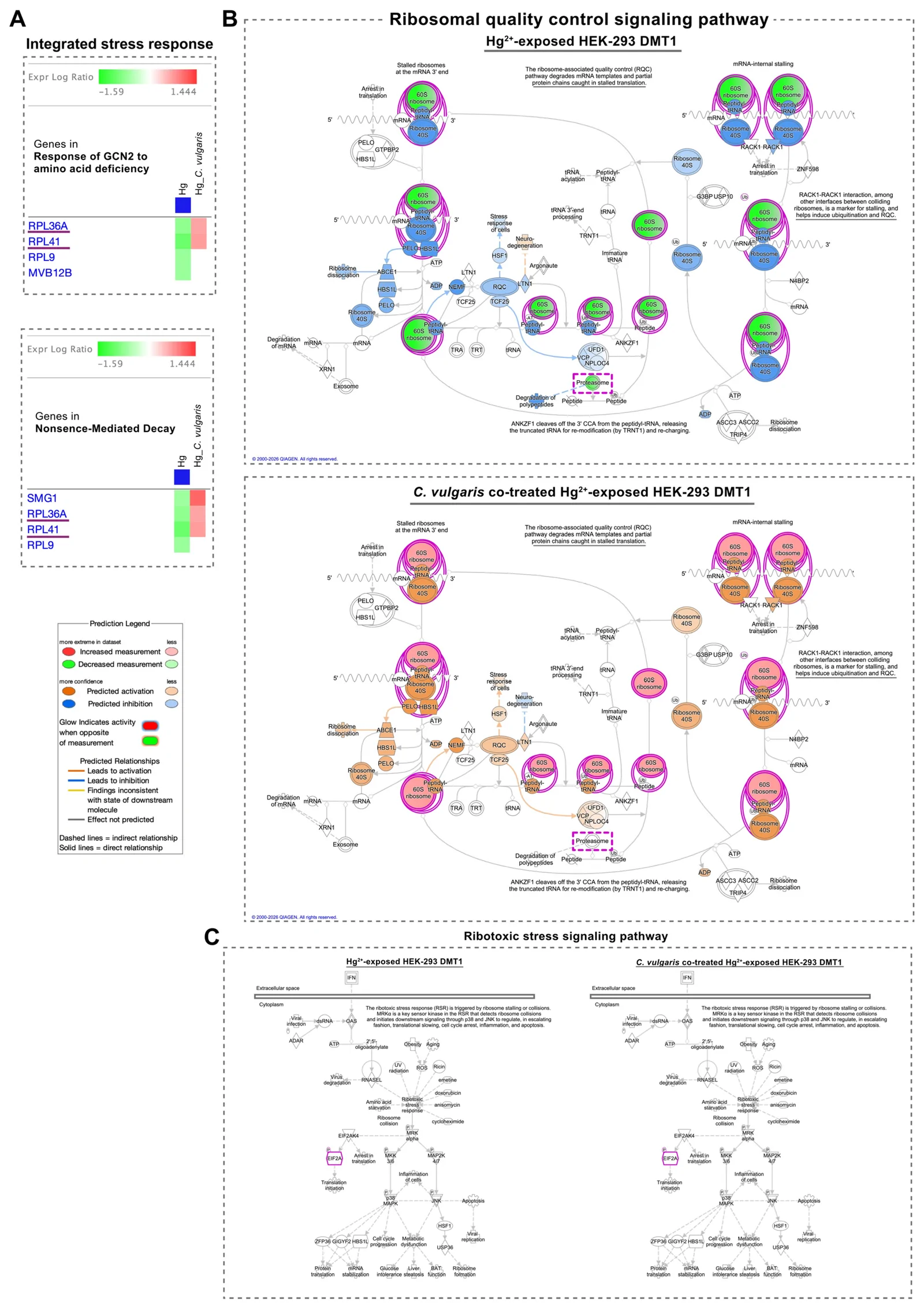
Mapping the ribosomal stress-surveillance pathways in Hg^2+^-exposed *versus C. vulgaris* co-treated HEK-293 DMT1 cells. (**A**) Gene expression heatmap of response of GCN2 to amino acid deficiency and nonsense-mediated decay pathways, (**B**) ribosome quality control signaling, and (**C**) ribotoxic stress response signaling between Hg^2+^-exposed and *C. vulgaris* co-treated HEK-293 DMT1 cells. The 60S ribosome components RPL36A and RPL41 are underlined in purple.

**Figure 12:**
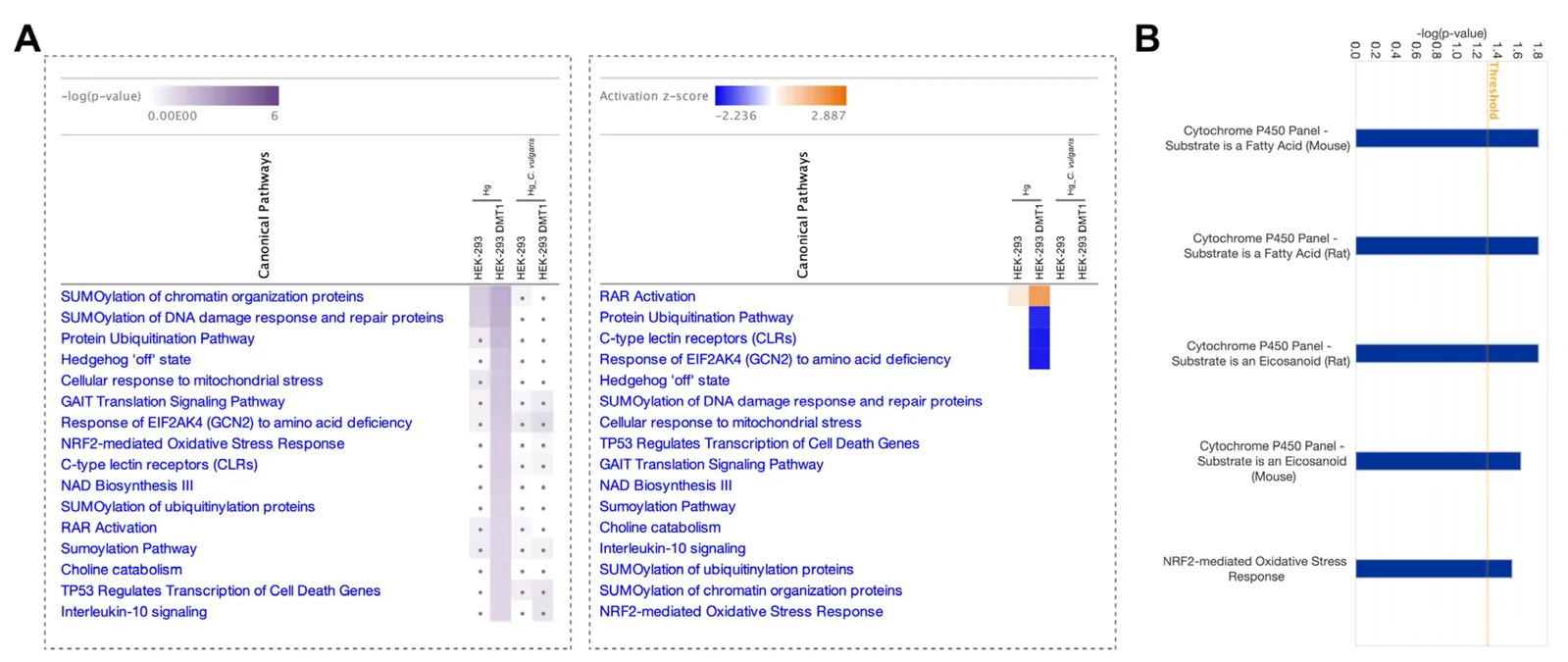
RAR activation signaling is activated through DMT1-mediated Hg^2+^ ion uptake. (**A**) IPA-based comparative canonical pathways analysis in Hg^2+^-exposed versus *C. vulgaris* co-treated HEK-293 and HEK-293 DMT1 cells. (**B**) Toxicity pathways generated by IPA in Hg2+-exposed HEK-293 DMT1 cells.

**Table 1:** IC_50_ values for HgCl_2_ in HEK-293 and HEK-293 DMT1 cells.

Cell Line	IC_50_ (μM)	95% CI	R^2^
HEK-293	3.84	2.43–6.09	0.95
HEK-293 DMT1	0.87	0.75–1.00	0.98

95% CI: asymptotic 95% confidence interval. Data were fitted with a four-parameter variable-slope model in which the lower plateau was constrained to 0% viability.

**Table 2:** Primer sequences of internal reference and eight deregulated genes used for qRT-PCR.

GENE Name	Gene Symbol	Forward Primer (5′-3′)	Reverse Primer (5′-3′)	Amplicon (bp)
Glyceraldehyde-3-phosphate dehydrogenase	*GAPDH*	GCTCTCTGCTCCTCCTGTTC	GACTCCGACCTTCACCTTCC	100
Solute carrier family 5 member 10	*SLC5A10*	TGCTCCAACATCGCCTACCC	GGAGGTCAGCGACGACATGA	108
Mesoderm development LRP chaperone	*MESD*	TCTGGCAGGGCAGCCTTTTC	AGTCCTTGATCTCCCAGGCG	114
Autophagy-related 16 like 1	*ATG16L1*	GTCCCGGACATGATGGCACA	CATCTCCCTGTCCTTCCGCT	164
Early growth response 1	*EGR1*	TTTCCTCACTCGCCCACCAT	TGGTCTCCACCAGCACCTTC	292
Establishment of sister chromatid cohesion N-acetyltransferase 2	*ESCO2*	GGAAAATCGTGTTGGTTCTGC	GCCTGTTTGATGGGTTCTGC	196
Nicotinamide nucleotide adenylyltransferase 2	*NMNAT2*	CCAGCACAGGGCTGCAAGTA	GTCGTGGACAGGGGAGACAA	189
Cell division cycle and apoptosis regulator 1	*CCAR1*	CAACCTCAGCAGGCCCTGTA	CCACCCCTGTGAAAACACGC	205
Transferrin receptor 2	*TFR2*	ACCCAGTTCCCTCCAGTTGC	AGGCTCCCCTGCCATTCTTG	131

## Data Availability

The original contributions presented in this study are included in the article/Supplementary Materials. Further inquiries can be directed to the corresponding author.

## Supplementary Materials

The following supporting information can be downloaded at: https://www.mdpi.com/article/10.3390/plants15192896/s1, Figure S1: Global quality assessment of the normalized microarray data; Table S1: Deregulated genes in Hg^2+^-exposed HEK-293 cells; Table S2: Deregulated genes in *C. vulgaris* co-treated Hg^2+^-exposed HEK-293 cells; Table S3: Deregulated genes in Hg^2+^-exposed HEK-293 DMT1 cells; Table S4: Deregulated genes in *C. vulgaris* co-treated Hg^2+^-exposed HEK-293 DMT1 cells.

## Abbreviations

BBB	Blood–brain barrier
CHMP2A	Charged multivesicular body protein 2A
DMT1	Divalent metal transporter 1
DEGs	Differentially expressed genes
DABG	Detection above background
DMPS	3-dimercapto-propanesulphonate
FADD	Fas-associated death domain protein
FDR	False discovery rate
GCN2/EIF2K4	Eukaryotic translation initiation factor 2 alpha kinase 4
HDAC10	Histone deacetylase 10
IPA	Ingenuity Pathway Analysis
IRPs	Iron regulatory proteins
KNL1	Kinetochore scaffold 1
LRP6	LDL receptor-related protein 6
MED21	Mediator complex subunit 21
NAC	N-acetyl-cysteine
NR2C2	Nuclear receptor subfamily 2 group C member 2
NSD2	Nuclear receptor binding SET domain protein 2
PSMD13	Proteasome 26S subunit, non-ATPase 13
RAR	Retinoic acid receptor
SD	Standard deviation
UBE2A	Ubiquitin-conjugating enzyme E2 A
UPLC-HRMS/MS	Ultra-performance liquid chromatography–high-resolution tandem mass spectrometry
