# Supplementary material for: The Protective Effect of Chlorella vulgaris Supplement Against Mercury Exposure Is Associated with DMT1: A Transcriptomic Analysis
Source: Plants (Basel). 2026 Sep 22;15(19):2896. doi: 10.3390/plants15192896 (PMC13667259; doi:10.3390/plants15192896)
Supplement: Supplementary file 1 [file plants-15-02896-s001.zip › plants-4499677-supplementary.pdf]

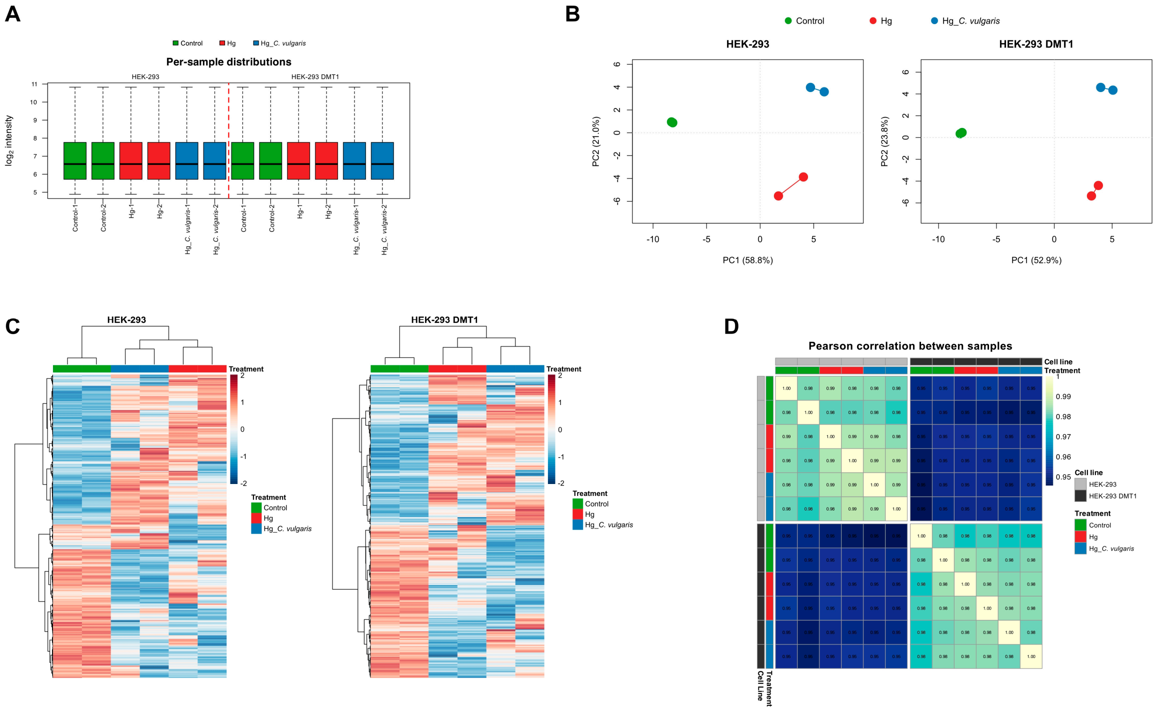

Supplementary Figure S1. Global quality assessment of the normalized microarray data. (A) Boxplot of per-sample intensity distributions across HEK-293 and HEK-293 DMT1 cells. Visualization of treatment signature in treatment-associated genes (nominal $p < 0.05$) across HEK-293 and HEK-293 DMT1 cells, using (B) principal component analysis (PCA) and (C) hierarchical clustering analysis. (D) Sample-to-sample Pearson correlation across HEK-293 and HEK-293 DMT1 cells.

Supplementary Table S1. Deregulated genes in Hg^{2+}-exposed HEK-293 cells

Symbol	Entrez Gene Name	Affymetrix Gene Code	Expr False Discovery Rate (q-value)	Expr Log Ratio
EGR1	early growth response 1	TC0500008785.hg.1	0	-1.378
MUC12	mucin 12, cell surface associated	TC0700008576.hg.1	0	-1.194
ATG16L1	autophagy related 16 like 1	TSUnmapped00000048.hg.1	0.000017	-1.009
SLU7	spliceosome associated SLU7	TC0500012663.hg.1	0.000088	-0.944
CLPTM1L	CLPTM1 like	TC0500009856.hg.1	0.000244	-0.905
COMMD9	COMM domain containing 9	TC1100010546.hg.1	0.00104	-0.852
KIAA0753	KIAA0753	TC1700009590.hg.1	0.00133	-0.837
MACF1	microtubule actin crosslinking factor 1	TC0100007898.hg.1	0.00249	-0.808
GARS1	glycyl-tRNA synthetase 1	TC0700007098.hg.1	0.00266	-0.804
TMEM14B	transmembrane protein 14B	TC0600014069.hg.1	0.00347	-0.792
PTDSS1	phosphatidylserine synthase 1	TC0800008295.hg.1	0.00424	-0.777
RET	ret proto-oncogene	TC1000007403.hg.1	0.00436	-0.775
POP1	POP1 ribonuclease P/MRP subunit	TC0800008330.hg.1	0.0056	-0.758
PGRMC2	progesterone receptor membrane component 2	TC0400011823.hg.1	0.0064	-0.751
PLA2G4D	phospholipase A2 group IVD	TC1500009179.hg.1	0.00714	-0.745
HDGFL2	HDGF like 2	TC1900006700.hg.1	0.00822	-0.736
NEDD1	NEDD1 gamma-tubulin ring complex targeting factor	TC1200008544.hg.1	0.00966	-0.727
FADD	Fas associated via death domain	TC1100008262.hg.1	0.00966	-0.726
LRP6	LDL receptor related protein 6	TSUnmapped00000166.hg.1	0.00966	-0.726
NIT2	nitrilase family member 2	TC0300008144.hg.1	0.0103	-0.723
ARID1B	AT-rich interaction domain 1B	TC0600009961.hg.1	0.0109	-0.718
CBL	Cbl proto-oncogene	TC1100009245.hg.1	0.0129	-0.708
FKBP1A-SDCBP2	FKBP1A-SDCBP2 readthrough (NMD candidate)	TC2000009966.hg.1	0.0132	-0.707
ROCK1	Rho associated coiled-coil containing protein kinase 1	TC1800008219.hg.1	0.0171	-0.693
RBM15B	RNA binding motif protein 15B	TC0300007438.hg.1	0.0171	-0.693
TMEM38B	transmembrane protein 38B	TC0900008318.hg.1	0.0172	-0.692
KIF14	kinesin family member 14	TC0100016891.hg.1	0.0202	-0.682
SPTB	spectrin beta, erythrocytic	TC1400009417.hg.1	0.0227	-0.677
AGRP	agouti related neuropeptide	TC1600010636.hg.1	0.0254	-0.665

LCOR	ligand dependent nuclear receptor corepressor	TC1000008558.hg.1	0.0271	-0.66
ARID4A	AT-rich interaction domain 4A	TC1400007307.hg.1	0.0276	-0.657
HIVEP2	HIVEP zinc finger 2	TC0600013409.hg.1	0.0276	-0.656
ELOA3BP (includes others)	elongin A3 family member B, pseudogene	TC1800008609.hg.1	0.0281	-0.655
DUSP7	dual specificity phosphatase 7	TC0300011152.hg.1	0.0362	-0.642
NSD2	nuclear receptor binding SET domain protein 2	TC0400006546.hg.1	0.0363	-0.642
RPRD1A	regulation of nuclear pre-mRNA domain containing 1A	TC1800008472.hg.1	0.0367	-0.641
BIRC6	baculoviral IAP repeat containing 6	TC0200007184.hg.1	0.037	-0.64
MARF1	meiosis regulator and mRNA stability factor 1	TC1600009530.hg.1	0.0388	-0.636
SURF4	surfeit 4	TSUnmapped00000076.hg.1	0.0388	-0.636
MDH1	malate dehydrogenase 1	TC0200007775.hg.1	0.0417	-0.632
DIS3L	DIS3 like exosome 3'-5' exoribonuclease	TC1500007612.hg.1	0.0438	-0.628
CCDC88A	coiled-coil and HOOK domain protein 88A	TC0200012643.hg.1	0.0438	-0.628
EID2	EP300 interacting inhibitor of differentiation 2	TC1900010665.hg.1	0.0448	-0.626
OMA1	OMA1 zinc metallopeptidase	TC0100018446.hg.1	0.0463	-0.625
FAHD1	fumarylacetoacetate hydrolase domain containing 1	TC1600006569.hg.1	0.0465	-0.624
GRAMD2A	GRAM domain containing 2A	TC1500009951.hg.1	0.0476	-0.622
EPIST	esophagus epithelial intergenic associated transcript	TC0500012094.hg.1	0.0488	-0.62
COQ8A	coenzyme Q8A	TC0100011812.hg.1	0.049	-0.619
DYNLL1	dynein light chain LC8-type 1	TC1200009131.hg.1	0.049	-0.618
UBN2	ubinuclein 2	TC0700009333.hg.1	0.049	-0.618
H2BC5	H2B clustered histone 5	TC0600007274.hg.1	0.049	-0.617
NPPC	natriuretic peptide C	TC0200016047.hg.1	0.049	-0.617
TM6SF1	transmembrane 6 superfamily member 1	TC1500008114.hg.1	0.0586	-0.606
C2CD3	C2 domain containing 3 centriole elongation regulator	TC1100011606.hg.1	0.0587	-0.605
TBC1D12	TBC1 domain family member 12	TC1000008502.hg.1	0.0595	-0.604
HDAC10	histone deacetylase 10	TC2200009360.hg.1	0.0632	-0.599
GMPS	guanine monophosphate synthase	TC0300009271.hg.1	0.0634	-0.598
CSNK1G2	casein kinase 1 gamma 2	TC1900006565.hg.1	0.0661	-0.596
ABHD12	abhydrolase domain containing 12, lysophospholipase	TC2000008741.hg.1	0.0683	-0.594
CRYBG3	crystallin beta-gamma domain containing 3	TC0300008088.hg.1	0.0711	-0.591

NSUN2	NOP2/Sun RNA methyltransferase 2	TC0500009996.hg.1	0.0722	-0.59
LIMK1	LIM domain kinase 1	TC0700007997.hg.1	0.0722	-0.59
SLMAP	sarcolemma associated protein	TC0300013841.hg.1	0.0781	-0.584
LGALS9B	galectin 9B	TC1700010050.hg.1	0.0787	-0.584
ATP5F1EP2	ATP synthase F1 subunit epsilon pseudogene 2	TC1300006699.hg.1	0.0787	-0.583
NOTUM	notum, palmitoleoyl-protein carboxylesterase	TC1700012084.hg.1	0.0795	-0.583
CCDC137	coiled-coil domain containing 137	TC1700009186.hg.1	0.083	-0.579
PRRC2A	proline rich coiled-coil 2A	TC0600007599.hg.1	0.0855	-0.577
EIF4G2	eukaryotic translation initiation factor 4 gamma 2	TC1100010092.hg.1	0.0872	-0.575
WDR7	WD repeat domain 7	TC1800007385.hg.1	0.0872	-0.575
AKAP17A	A-kinase anchoring protein 17A	TC0X00006460.hg.1	0.0917	-0.57
SLC16A1	solute carrier family 16 member 1	TSUnmapped00000763.hg.1	0.0925	-0.569
FOXC2	forkhead box C2	TC1600008738.hg.1	0.0925	-0.568
EOMES	eomesodermin	TC0300010566.hg.1	0.0945	-0.567
OARD1	O-acyl-ADP-ribose deacylase 1	TC0600014292.hg.1	0.0937	-0.567
RAX2	retina and anterior neural fold homeobox 2	TC1900009308.hg.1	0.0945	-0.566
RSAD2	radical S-adenosyl methionine domain containing 2	TC0200016402.hg.1	0.0966	-0.565
GLDC	glycine decarboxylase	TC0900009489.hg.1	0.0973	-0.564
FAM25EP	family with sequence similarity 25 member E	TC1000010507.hg.1	0.0973	-0.564
PLXNA2	plexin A2	TC0100017145.hg.1	0.0991	-0.56
NCKAP1	NCK associated protein 1	TC0200016757.hg.1	0.0991	-0.56
MFSD2A	MFSD2 lysolipid transporter A, lysophospholipid	TC0100007936.hg.1	0.0991	-0.56
NCAN	neurocan	TC1900007426.hg.1	0.0999	-0.559
DNAH14	dynein axonemal heavy chain 14	TC0100011755.hg.1	0.0995	0.559
ZNF627	zinc finger protein 627	TC1900011666.hg.1	0.0991	0.56
MESD	mesoderm development LRP chaperone	TC1500010907.hg.1	0.0985	0.563
COL19A1	collagen type XIX alpha 1 chain	TC0600008462.hg.1	0.0973	0.564
PPFIBP1	PPFIB scaffold protein 1	TC1200007153.hg.1	0.0947	0.566
STC2	stanniocalcin 2	TC0500012870.hg.1	0.0925	0.568
HYAL1	hyaluronidase 1	TC0300011103.hg.1	0.0925	0.568
SATB1	SATB homeobox 1	TC0300013949.hg.1	0.0925	0.569
MRNIP	MRN complex interacting protein	TC0500013425.hg.1	0.0925	0.569
HERPUD1	homocysteine inducible ER protein with ubiquitin like domain 1	TC1600007982.hg.1	0.0924	0.57
UTP23	UTP23 small subunit processome component	TC0800008626.hg.1	0.0891	0.572
CWC15	CWC15 spliceosome associated protein	TC1100012012.hg.1	0.0891	0.572
KIF18B	kinesin family member 18B	TC1700010851.hg.1	0.0891	0.572

RPL7A	ribosomal protein L7a	TSUnmapped00000053.hg.1	0.0875	0.574
HOXA3	homeobox A3	TC0700013529.hg.1	0.088	0.574
ZNF670	zinc finger protein 670	TC0100018081.hg.1	0.0872	0.575
PIF1	PIF1 5'-to-3' DNA helicase	TC1500009760.hg.1	0.0872	0.575
TRMT1L	tRNA methyltransferase 1L	TC0100016685.hg.1	0.0872	0.575
CELA2A	chymotrypsin like elastase 2A	TC0100018177.hg.1	0.0872	0.576
UBLCP1	ubiquitin like domain containing CTD phosphatase 1	TC0500009250.hg.1	0.0872	0.576
THAP5	THAP domain containing 5	TC0700013606.hg.1	0.0819	0.58
SIGIRR	single Ig and TIR domain containing	TC1100009666.hg.1	0.0819	0.58
ZNF726	zinc finger protein 726	TC1900007619.hg.1	0.0818	0.581
RPF1	ribosome production factor 1 homolog	TC0100008884.hg.1	0.0795	0.582
ZNF780B	zinc finger protein 780B	TSUnmapped00000100.hg.1	0.0795	0.583
CHMP2A	charged multivesicular body protein 2A	TC1900011635.hg.1	0.0787	0.584
INSIG2	insulin induced gene 2	TC0200009049.hg.1	0.0781	0.585
PHF20L1	PHD finger protein 20 like 1	TC0800008940.hg.1	0.0742	0.588
ELOVL4	ELOVL fatty acid elongase 4	TC0600012379.hg.1	0.0726	0.589
DDX31	DEAD-box helicase 31	TC0900011811.hg.1	0.0719	0.59
TMEM208	transmembrane protein 208	TC1600008153.hg.1	0.0717	0.591
ZNF875	zinc finger protein 875	TC1900011722.hg.1	0.0694	0.593
NRSN2-AS1	NRSN2 antisense RNA 1	TC2000008123.hg.1	0.067	0.595
ZNF492/ZNF98	zinc finger protein 492	TC1900007570.hg.1	0.0641	0.597
CENPK	centromere protein K	TC0500010928.hg.1	0.0634	0.598
LIG4	DNA ligase 4	TC1300009740.hg.1	0.0627	0.6
COPRS	coordinator of PRMT5 and differentiation stimulator	TC1700010327.hg.1	0.0615	0.601
ZNF578	zinc finger protein 578	TC1900008702.hg.1	0.061	0.601
ZNF321P	zinc finger protein 321, pseudogene	TC1900012030.hg.1	0.0609	0.602
CLIP1	CAP-Gly domain containing linker protein 1	TC1200012234.hg.1	0.0587	0.604
MTHFSD	methenyltetrahydrofolate synthetase domain containing	TC1600011147.hg.1	0.0587	0.605
S100A1	S100 calcium binding protein A1	TC0100010043.hg.1	0.0587	0.605
FAM76B	family with sequence similarity 76 member B	TC1100012029.hg.1	0.0587	0.605
PNMA5	PNMA family member 5	TC0X00011144.hg.1	0.0587	0.606
AASDH	aminoadipate-semialdehyde dehydrogenase	TC0400010768.hg.1	0.0586	0.606
PROP1	PROP paired-like homeobox 1	TC0500013002.hg.1	0.0569	0.608
MED21	mediator complex subunit 21	TC1200012612.hg.1	0.056	0.609
GNA15	G protein subunit alpha 15	TC1900006620.hg.1	0.0554	0.61

NR6A1	nuclear receptor subfamily 6 group A member 1	TC0900011501.hg.1	0.0533	0.612
ENPP5	ectonucleotide pyrophosphatase/phosphodiesterase family member 5	TC0600011943.hg.1	0.0523	0.613
MSGN1	mesogenin 1	TC0200006835.hg.1	0.0508	0.615
CACNA2D3	calcium voltage-gated channel auxiliary subunit alpha2delta 3	TC0300007529.hg.1	0.049	0.617
ZNF586	zinc finger protein 586	TC1900011822.hg.1	0.049	0.619
XRCC5	X-ray repair cross complementing 5	TC0200010729.hg.1	0.0488	0.619
HMMR	hyaluronan mediated motility receptor	TC0500009320.hg.1	0.0488	0.62
ACTR6	actin related protein 6	TC1200008591.hg.1	0.0468	0.623
CATSPERE	catsper channel auxiliary subunit epsilon	TC0100012221.hg.1	0.0463	0.624
ZNF217	zinc finger protein 217	TC2000009504.hg.1	0.0438	0.628
NAGLU	N-acetyl-alpha-glucosaminidase	TC1700012259.hg.1	0.0431	0.63
TAF15	TATA-box binding protein associated factor 15	TC1700007606.hg.1	0.0422	0.632
STXBP3	syntaxin binding protein 3	TC0100009331.hg.1	0.0388	0.636
H2BC21	H2B clustered histone 21	TC0100018483.hg.1	0.0388	0.637
DLX5	distal-less homeobox 5	TC0700011865.hg.1	0.0379	0.638
SAG	S-antigen visual arrestin	TSUnmapped00000152.hg.1	0.0374	0.639
U2AF1L4	U2 small nuclear RNA auxiliary factor 1 like 4	TC1900011930.hg.1	0.0341	0.645
OR4D2	olfactory receptor family 4 subfamily D member 2	TC1700008423.hg.1	0.0317	0.649
NCR3LG1	natural killer cell cytotoxicity receptor 3 ligand 1	TC1100006966.hg.1	0.0316	0.649
KNL1	kinetochore scaffold 1	TC1500006963.hg.1	0.0287	0.654
TBC1D8B	TBC1 domain family member 8B	TC0X00008055.hg.1	0.0276	0.657
SMC5	structural maintenance of chromosomes 5	TC0900007520.hg.1	0.0276	0.658
CBX5	chromobox 5	TC1200010833.hg.1	0.0271	0.659
HES6	hes family bHLH transcription factor 6	TC0200016222.hg.1	0.0271	0.66
PRKAR1A	protein kinase cAMP-dependent type I regulatory subunit alpha	TC1700012296.hg.1	0.0263	0.662
SLC2A14	solute carrier family 2 member 14	TC1200009796.hg.1	0.0255	0.664
KLHL11	kelch like family member 11	TC1700010692.hg.1	0.0252	0.665
MZT1	mitotic spindle organizing protein 1	TC1300009229.hg.1	0.0252	0.666
RRP36	ribosomal RNA processing 36	TC0600014129.hg.1	0.0252	0.666
TMEM234	transmembrane protein 234	TC0100013602.hg.1	0.0252	0.667
H3C1	H3 clustered histone 1	TC0600007262.hg.1	0.0238	0.67
PRMT6	protein arginine methyltransferase 6	TC0100009305.hg.1	0.0238	0.67
SEMA3C	semaphorin 3C	TC0700011626.hg.1	0.024	0.67
UBE3A	ubiquitin protein ligase E3A	TC1500008751.hg.1	0.0233	0.672
DGLUCY	D-glutamate cyclase	TC1400007975.hg.1	0.0233	0.672

NTAQ1	N-terminal glutamine amidase 1	TC0800012351.hg.1	0.0232	0.673
LETMD1	LETM1 domain containing 1	TC1200007630.hg.1	0.0227	0.674
OSBPL9	oxysterol binding protein like 9	TC0100008289.hg.1	0.0227	0.675
COL2A1	collagen type II alpha 1 chain	TC1200010562.hg.1	0.0227	0.676
GOLGA8K (includes others)	golgin A8 family member K	TC1500006699.hg.1	0.0197	0.684
SENP5	SUMO specific peptidase 5	TC0300010038.hg.1	0.0187	0.686
ZNF585A	zinc finger protein 585A	TC1900011933.hg.1	0.0188	0.686
KIF27	kinesin family member 27	TC0900010582.hg.1	0.0185	0.687
ZNF226	zinc finger protein 226	TC1900011755.hg.1	0.0183	0.688
FASTKD3	FAST kinase domains 3	TC0500010019.hg.1	0.0174	0.691
MMACHC	metabolism of cobalamin associated C	TC0100008124.hg.1	0.0174	0.691
APOBEC3F	apolipoprotein B mRNA editing enzyme catalytic subunit 3F	TC2200009273.hg.1	0.0159	0.697
LIAS	lipoic acid synthetase	TC0400007285.hg.1	0.0159	0.698
PHC3	polyhomeotic homolog 3	TC0300013090.hg.1	0.0123	0.711
GNG4	G protein subunit gamma 4	TC0100017834.hg.1	0.0103	0.722
RTL8A	retrotransposon Gag like 8A	TC0X00011399.hg.1	0.0084	0.734
ABCB4	ATP binding cassette subfamily B member 4	TC0700011705.hg.1	0.00768	0.74
ZNF567	zinc finger protein 567	TC1900007950.hg.1	0.00714	0.744
RAD52	RAD52 DNA repair protein	TC1200009566.hg.1	0.00714	0.744
SEC23B	SEC23 homolog B, COPII component	TC2000006833.hg.1	0.00539	0.763
ZFAT	zinc finger and AT-hook domain containing	TC0800011907.hg.1	0.00514	0.766
VPS26A	VPS26 retromer complex component A	TC1000007888.hg.1	0.00514	0.767
NPAS1	neuronal PAS domain protein 1	TC1900008404.hg.1	0.00411	0.782
LIN9	lin-9 DREAM MuvB core complex component	TC0100017523.hg.1	0.00376	0.788
PLSCR1	phospholipid scramblase 1	TC0300012728.hg.1	0.00303	0.798
USP45	ubiquitin specific peptidase 45	TC0600012656.hg.1	0.00248	0.809
KLF9	KLF transcription factor 9	TC0900010366.hg.1	0.00179	0.822
SLX4	SLX4 structure-specific endonuclease subunit	TC1600009199.hg.1	0.00141	0.833
PEAK1	pseudopodium enriched atypical kinase 1	TC1500010109.hg.1	0.00133	0.838
CTTNBP2NL	CTTNBP2 N-terminal like	TC0100009441.hg.1	0.00133	0.839
EBF3	EBF transcription factor 3	TC1000012270.hg.1	0.00127	0.843
ADH5	alcohol dehydrogenase 5 (class III), chi polypeptide	TC0400011402.hg.1	0.000552	0.875
H2BC26	H2B clustered histone 26	TC0100011871.hg.1	0.000204	0.913
SHANK3	SH3 and multiple ankyrin repeat domains 3	TC2200007825.hg.1	0.000164	0.922
MT-ND2	NADH dehydrogenase subunit 2	TC0M00006434.hg.1	0.000058	0.959

VRK1	VRK serine/threonine kinase 1	TC1400010649.hg.1	0.000056	0.963
SLC5A10	solute carrier family 5 member 10	TC1700007145.hg.1	0.00002	1
NR2C2	nuclear receptor subfamily 2 group C member 2	TC0300006696.hg.1	0.000017	1.01
CLPTM1	CLPTM1 regulator of GABA type A receptor forward trafficking	TC1900008297.hg.1	0.000004	1.055
RPL36A	ribosomal protein L36a	TC0X00011308.hg.1	0	1.283

© 2000-2026 QIAGEN. All rights reserved.

Supplementary Table S2. Deregulated genes in *C. vulgaris* co-treated Hg^{2+}-exposed HEK-293 cells

Symbol	Entrez Gene Name	Affymetrix Gene Code	Expr False Discovery Rate (q-value)	Expr Log Ratio
H2AC25	H2A clustered histone 25	TC0100017617.hg.1	0	-1.331
RTL8A	retrotransposon Gag like 8A	TC0X00011399.hg.1	0	-1.243
ZNF429	zinc finger protein 429	TC1900011692.hg.1	0.000022	-0.943
GLOD4	glyoxalase domain containing 4	TC1700012322.hg.1	0.000041	-0.924
ARHGEF19	Rho guanine nucleotide exchange factor 19	TC0100013034.hg.1	0.000165	-0.878
H2BC11	H2B clustered histone 11	TC0600011184.hg.1	0.000847	-0.807
MT-ND2	NADH dehydrogenase subunit 2	TC0M00006434.hg.1	0.00179	-0.773
UQCC2	ubiquinol-cytochrome c reductase complex assembly factor 2	TC0600011560.hg.1	0.00366	-0.736
MIEN1	migration and invasion enhancer 1	TC1700010584.hg.1	0.00444	-0.727
UQCRQ	ubiquinol-cytochrome c reductase complex III subunit VII	TC0500008653.hg.1	0.00554	-0.716
ETV7	ETS variant transcription factor 7	TC0600011658.hg.1	0.00554	-0.716
GNG4	G protein subunit gamma 4	TC0100017834.hg.1	0.00562	-0.714
CAV1	caveolin 1	TC0700008873.hg.1	0.00919	-0.695
SKI	SKI proto-oncogene	TC0100006565.hg.1	0.0109	-0.687
CDH16	cadherin 16	TC1600010602.hg.1	0.0112	-0.683
ZNF567	zinc finger protein 567	TC1900007950.hg.1	0.0112	-0.683
SENP6	SUMO specific peptidase 6	TC0600008562.hg.1	0.0166	-0.665
CLASRP	CLK4 associating serine/arginine rich protein	TC1900008303.hg.1	0.0175	-0.66
PHF8	PHD finger protein 8	TC0X00009773.hg.1	0.0175	-0.659
EDA	ectodysplasin A	TC0X00007523.hg.1	0.0175	-0.659

GPAM	glycerol-3-phosphate acyltransferase, mitochondrial	TC1000011839.hg.1	0.0185	-0.656
DRG2	developmentally regulated GTP binding protein 2	TC1700007099.hg.1	0.0188	-0.655
CHMP2A	charged multivesicular body protein 2A	TC1900011635.hg.1	0.0197	-0.652
POMP	proteasome maturation protein	TC1300006729.hg.1	0.0223	-0.644
H4C4	H4 clustered histone 4	TC0600011134.hg.1	0.0238	-0.64
IFFO1	intermediate filament family orphan 1	TC1200009740.hg.1	0.025	-0.636
RPL9P7	ribosomal protein L9 pseudogene 7	TC0X00009255.hg.1	0.0252	-0.635
TNFRSF10C	TNF receptor superfamily member 10c	TC0800007007.hg.1	0.0285	-0.628
CNEP1R1	CTD nuclear envelope phosphatase 1 regulatory subunit 1	TC1600007803.hg.1	0.0285	-0.627
VPS26A	VPS26 retromer complex component A	TC1000007888.hg.1	0.0285	-0.627
RPL9	ribosomal protein L9	TC1500010237.hg.1	0.0309	-0.621
LIAS	lipoic acid synthetase	TC0400007285.hg.1	0.0319	-0.619
HYOU1	hypoxia up-regulated 1	TSUnmapped00000440.hg.1	0.0329	-0.616
IHH	Indian hedgehog signaling molecule	TC0200015759.hg.1	0.0386	-0.609
MED21	mediator complex subunit 21	TC1200012612.hg.1	0.0386	-0.608
ZNF322	zinc finger protein 322	TC0600011162.hg.1	0.0394	-0.607
NTAN1	N-terminal asparagine amidase	TC1600009512.hg.1	0.0412	-0.605
TXNDC17	thioredoxin domain containing 17	TC1700006702.hg.1	0.0418	-0.603
DUSP5	dual specificity phosphatase 5	TC1000008891.hg.1	0.0418	-0.603
BRPF3	bromodomain and PHD finger containing 3	TC0600007832.hg.1	0.0429	-0.601

MMACHC	metabolism of cobalamin associated C	TC0100008124.hg.1	0.0427	-0.601
HAP1	huntingtin associated protein 1	TC1700010684.hg.1	0.0434	-0.6
ZNF92	zinc finger protein 92	TC0700007807.hg.1	0.0446	-0.598
EXOC2	exocyst complex component 2	TC0600010375.hg.1	0.0446	-0.597
SLC4A4	solute carrier family 4 member 4	TC0400007799.hg.1	0.0446	-0.597
SYNE1	spectrin repeat containing nuclear envelope protein 1	TC0600013588.hg.1	0.0489	-0.592
CHRNA4	cholinergic receptor nicotinic alpha 4 subunit	TC2000010033.hg.1	0.0489	-0.592
UBXN2B	UBX domain protein 2B	TC0800007735.hg.1	0.0502	-0.59
IL17C	interleukin 17C	TC1600008832.hg.1	0.0501	-0.59
SAMM50	SAMM50 sorting and assembly machinery component	TC2200009282.hg.1	0.0598	-0.58
SETDB2	SET domain bifurcated histone lysine methyltransferase 2	TC1300007164.hg.1	0.0664	-0.574
ABCB4	ATP binding cassette subfamily B member 4	TC0700011705.hg.1	0.0665	-0.574
CTTNBP2NL	CTTNBP2 N-terminal like	TC0100009441.hg.1	0.0678	-0.572
MAGEA12	MAGE family member A12	TC0X00008752.hg.1	0.0678	-0.571
DNAH14	dynein axonemal heavy chain 14	TC0100011755.hg.1	0.0713	-0.568
LANCL1	LanC like glutathione S-transferase 1	TC0200015608.hg.1	0.0731	-0.567
NR2C2	nuclear receptor subfamily 2 group C member 2	TC0300006696.hg.1	0.0735	-0.566
KNL1	kinetochore scaffold 1	TC1500006963.hg.1	0.0742	-0.565
RPS4Y1	ribosomal protein S4 Y-linked 1	TC0Y00007322.hg.1	0.077	-0.563
TMEM87A	transmembrane protein 87A	TC1500009185.hg.1	0.077	-0.563
TLNRD1	talin rod domain containing 1	TC1500008052.hg.1	0.0877	-0.554

ASTE1	asteroid structure-specific endonuclease 1	TC0300012435.hg.1	0.0937	-0.55
CAV2	caveolin 2	TC0700008870.hg.1	0.0945	0.549
SYAP1	synapse associated protein 1	TC0X00006697.hg.1	0.0937	0.55
ZBTB46	zinc finger and BTB domain containing 46	TC2000009833.hg.1	0.0908	0.552
ZNF208	zinc finger protein 208	TC1900010164.hg.1	0.081	0.558
SLC16A1	solute carrier family 16 member 1	TSUnmapped00000763.hg.1	0.0814	0.558
ZYX	zyxin	TC0700009493.hg.1	0.0786	0.56
LPP	LIM domain containing preferred translocation partner in lipoma	TC0300009815.hg.1	0.0786	0.56
HLA-DRB5	major histocompatibility complex, class II, DR beta 5	TC0600011491.hg.1	0.0786	0.561
DUSP16	dual specificity phosphatase 16	TSUnmapped00000144.hg.1	0.0786	0.561
TRIP11	thyroid hormone receptor interactor 11	TC1400010778.hg.1	0.0785	0.561
CACFD1	calcium channel flower domain containing 1	TSUnmapped00000519.hg.1	0.0742	0.565
DYNC2LI1	dynein cytoplasmic 2 light intermediate chain 1	TC0200007401.hg.1	0.0735	0.566
AHSA2P	activator of HSP90 ATPase homolog 2, pseudogene	TC0200007717.hg.1	0.0709	0.568
CYP11B1	cytochrome P450 family 11 subfamily B member 1	TC0800012092.hg.1	0.0664	0.575
POGZ	pogo transposable element derived with ZNF domain	TC0100015791.hg.1	0.0629	0.577
TMEM132A	transmembrane protein 132A	TC1100007786.hg.1	0.057	0.582
ST6GALNAC3	ST6 N-acetylgalactosaminide alpha-2,6-sialyltransferase 3	TC0100008779.hg.1	0.0563	0.583

NSD2	nuclear receptor binding SET domain protein 2	TC0400006546.hg.1	0.0549	0.585
NDUFA10	NADH:ubiquinone oxidoreductase subunit A10	TSUnmapped00000163.hg.1	0.0522	0.587
MORN3	MORN repeat containing 3	TC1200012210.hg.1	0.0501	0.59
LMO1	LIM domain only 1	TC1100010026.hg.1	0.0489	0.592
C1QTNF3-AMACR	C1QTNF3-AMACR readthrough (NMD candidate)	TC0500013296.hg.1	0.0486	0.593
H3C13	H3 clustered histone 13	TC0100015698.hg.1	0.0463	0.595
POLR3D	RNA polymerase III subunit D	TC0800006978.hg.1	0.0442	0.599
CPE	carboxypeptidase E	TC0400009223.hg.1	0.0434	0.6
GAS2L1	growth arrest specific 2 like 1	TC2200007014.hg.1	0.0418	0.603
CFAP61	cilia and flagella associated protein 61	TC2000006868.hg.1	0.0404	0.606
KAT2A	lysine acetyltransferase 2A	TC1700012415.hg.1	0.0361	0.612
FKBP1A-SDCBP2	FKBP1A-SDCBP2 readthrough (NMD candidate)	TC2000009966.hg.1	0.0328	0.617
FHIP1A	FHF complex subunit HOOK interacting protein 1A	TC0400009001.hg.1	0.0319	0.618
TMEM106B	transmembrane protein 106B	TC0700006727.hg.1	0.0308	0.621
STOX2	storkhead box 2	TC0400012868.hg.1	0.0309	0.621
DMXL1	Dmx like 1	TC0500008431.hg.1	0.0308	0.622
SNCG	synuclein gamma	TC1000008320.hg.1	0.0298	0.623
LRRC34	leucine rich repeat containing 34	TC0300013083.hg.1	0.0297	0.624
ARRB1	arrestin beta 1	TC1100011643.hg.1	0.0297	0.624
IRX2	iroquois homeobox 2	TC0500009909.hg.1	0.0297	0.624
FGFR1OP2	FGFR1 oncogene partner 2	TC1200007137.hg.1	0.0285	0.627
MLXIP	MLX interacting protein	TSUnmapped00000502.hg.1	0.0257	0.634
ABR	ABR activator of RhoGEF and GTPase	TC1700012324.hg.1	0.0249	0.636
LILRA5	leukocyte immunoglobulin like receptor A5	TC1900012040.hg.1	0.0249	0.637

PPME1	protein phosphatase methylesterase 1	TC1100008409.hg.1	0.0249	0.637
TBCEL	tubulin folding cofactor E like	TC1100009301.hg.1	0.0244	0.639
DEGS2	delta 4-desaturase, sphingolipid 2	TC1400010182.hg.1	0.0238	0.64
PPP1R26	protein phosphatase 1 regulatory subunit 26	TC0900009163.hg.1	0.0238	0.64
RAB31	RAB31, member RAS oncogene family	TC1800006650.hg.1	0.0223	0.644
SLC38A6	solute carrier family 38 member 6	TC1400010616.hg.1	0.0209	0.648
UBN2	ubinuclein 2	TC0700009333.hg.1	0.02	0.65
GAD2	glutamate decarboxylase 2	TC1000007068.hg.1	0.0197	0.652
FADD	Fas associated via death domain	TC1100008262.hg.1	0.0197	0.652
PTGIR	prostaglandin I2 receptor	TC1900010994.hg.1	0.0197	0.653
GTPBP1	GTP binding protein 1	TC2200009267.hg.1	0.0175	0.66
PLEKHG4B	pleckstrin homology and RhoGEF domain containing G4B	TC0500006435.hg.1	0.0175	0.66
TAGAP	T cell activation RhoGTPase activating protein	TC0600013743.hg.1	0.017	0.663
TAS2R31	taste 2 receptor member 31	TC1200012761.hg.1	0.0166	0.664
ZNF641	zinc finger protein 641	TC1200010577.hg.1	0.0163	0.666
ANKRD18A	ankyrin repeat domain 18A	TC0900010056.hg.1	0.0159	0.668
STX16-NPEPL1	STX16-NPEPL1 readthrough (NMD candidate)	TC2000009947.hg.1	0.0115	0.681
TMEM38B	transmembrane protein 38B	TC0900008318.hg.1	0.0112	0.683
TTC24	tetratricopeptide repeat domain 24	TC0100010184.hg.1	0.011	0.686
ZNF268	zinc finger protein 268	TC1200012737.hg.1	0.0109	0.688
RAD54L	RAD54 like	TC0100008150.hg.1	0.00579	0.713
POLR2A	RNA polymerase II subunit A	TC1700006751.hg.1	0.005	0.721
PLA2G4F	phospholipase A2 group IVF	TC1500009181.hg.1	0.00444	0.727

L3HYPDH	trans-L-3-hydroxyproline dehydratase	TC1400010757.hg.1	0.00406	0.732
TBC1D3 (includes others)	TBC1 domain family member 3	TC1700012241.hg.1	0.00366	0.737
MUC12	mucin 12, cell surface associated	TC0700008576.hg.1	0.00366	0.738
KATNIP	katanin interacting protein	TC1600007317.hg.1	0.00304	0.746
ZNF780B	zinc finger protein 780B	TSUnmapped00000206.hg.1	0.0029	0.748
NIT2	nitrilase family member 2	TC0300008144.hg.1	0.0029	0.749
SMIM11	small integral membrane protein 11	TC2100008499.hg.1	0.0028	0.751
KPNA3	karyopherin subunit alpha 3	TC1300008927.hg.1	0.00257	0.755
ATG16L1	autophagy related 16 like 1	TSUnmapped00000048.hg.1	0.00252	0.756
ADGRL3	adhesion G protein-coupled receptor L3	TC0400012797.hg.1	0.00233	0.76
FAHD1	fumarylacetoacetate hydrolase domain containing 1	TC1600006569.hg.1	0.002	0.766
BRI3BP	BRI3 binding protein	TC1200009300.hg.1	0.00179	0.772
SLMAP	sarcolemma associated protein	TC0300013841.hg.1	0.00129	0.786
FAM25EP	family with sequence similarity 25 member E	TC1000010507.hg.1	0.00114	0.793
NUBPL	NUBP iron-sulfur cluster assembly factor, mitochondrial	TC1400010592.hg.1	0.00105	0.798
ZDHHC3	zDHHC palmitoyltransferase 3	TSUnmapped00000569.hg.1	0.00103	0.8
LRP6	LDL receptor related protein 6	TSUnmapped00000166.hg.1	0.000847	0.807
OSMR	oncostatin M receptor	TC0500007201.hg.1	0.000779	0.816
OARD1	O-acyl-ADP-ribose deacylase 1	TC0600014292.hg.1	0.000662	0.822
TBC1D12	TBC1 domain family member 12	TC1000008502.hg.1	0.000662	0.823
TMEM175	transmembrane protein 175	TC0400006487.hg.1	0.000649	0.826
KIAA0753	KIAA0753	TC1700009590.hg.1	0.000473	0.838

WNK1	WNK lysine deficient protein kinase 1	TC1200006454.hg.1	0.000224	0.865
SLU7	spliceosome associated SLU7	TC0500012663.hg.1	0.000091	0.898
HDAC10	histone deacetylase 10	TC2200009360.hg.1	0.000006	0.99
PBRM1	polybromo 1	TC0300011175.hg.1	0	1.154
HNRNPH2	heterogeneous nuclear ribonucleoprotein H2	TC0X00011309.hg.1	0	1.303

© 2000-2026 QIAGEN. All rights reserved.

Supplementary Table S3. Deregulated genes in Hg^{2+}-exposed HEK-293 DMT1 cells

Symbol	Entrez Gene Name	Affymetrix Gene Code	Expr False Discovery Rate (q-value)	Expr Log Ratio
CCAR1	cell division cycle and apoptosis regulator 1	TC1000007866.hg.1	0	-1.59
RPL41	ribosomal protein L41	TC2200008578.hg.1	0.000016	-1.052
PCDHB7	protocadherin beta 7	TC0500008880.hg.1	0.000034	-1.018
SENP6	SUMO specific peptidase 6	TC0600008562.hg.1	0.000041	-1.005
SCAF8	SR-related CTD associated factor 8	TC0600014193.hg.1	0.000059	-0.991
ZNF205	zinc finger protein 205	TC1600006664.hg.1	0.000067	-0.985
ANTKMT	adenine nucleotide translocase lysine methyltransferase	TC1600006493.hg.1	0.000124	-0.957
ICAM1	intercellular adhesion molecule 1	TC1900006977.hg.1	0.000457	-0.904
UQCRB	ubiquinol-cytochrome c reductase binding protein	TC0800011171.hg.1	0.000564	-0.896
GPR21	G protein-coupled receptor 21	TC0900008684.hg.1	0.000901	-0.874
LY6E	lymphocyte antigen 6 family member E	TC0800012372.hg.1	0.000985	-0.869
IFNL1	interferon lambda 1	TC1900008051.hg.1	0.00115	-0.861
WDR83OS	WD repeat domain 83 opposite strand	TC1900011890.hg.1	0.00166	-0.843
SCN8A	sodium voltage-gated channel alpha subunit 8	TC1200010711.hg.1	0.0018	-0.838
MUC12	mucin 12, cell surface associated	TC0700008576.hg.1	0.0018	-0.838
LYAR	Ly1 antibody reactive	TC0400009856.hg.1	0.002	-0.834
TNFRSF10C	TNF receptor superfamily member 10c	TC0800007007.hg.1	0.00219	-0.829
RBM4B	RNA binding motif protein 4B	TC1100011299.hg.1	0.00314	-0.81
LLPH	LLP homolog, long-term synaptic facilitation factor	TC1200012818.hg.1	0.00367	-0.804
BMERB1	bMERB domain containing 1	TC1600011358.hg.1	0.00367	-0.803
RPL36A	ribosomal protein L36a	TC0X00011308.hg.1	0.00429	-0.795
PUDP	pseudouridine 5'-phosphatase	TC0X00009007.hg.1	0.00736	-0.768
TFR2	transferrin receptor 2	TC0700012010.hg.1	0.00736	-0.767
PSMD13	proteasome 26S subunit, non-ATPase 13	TC1100006441.hg.1	0.00739	-0.767
NKX6-1	NK6 homeobox 1	TC0400011217.hg.1	0.00783	-0.761

CNGA3	cyclic nucleotide gated channel subunit alpha 3	TC0200008566.hg.1	0.00794	-0.76
CLIC3	chloride intracellular channel 3	TC0900012024.hg.1	0.0095	-0.752
CLEC2D	C-type lectin domain family 2 member D	TC1200006771.hg.1	0.0108	-0.746
RASSF10	Ras association domain family member 10	TC1100006896.hg.1	0.0118	-0.741
SLC16A1	solute carrier family 16 member 1	TSUnmapped00000178 .hg.1	0.0122	-0.739
SLC22A7	solute carrier family 22 member 7	TC0600008078.hg.1	0.0124	-0.737
GPC3	glypican 3	TC0X00010851.hg.1	0.0124	-0.736
BBLN	bublin coiled coil protein	TC0900008849.hg.1	0.0125	-0.735
SLC25A4 1	solute carrier family 25 member 41	TC1900009430.hg.1	0.0144	-0.726
PCDHB9	protocadherin beta 9	TC0500013245.hg.1	0.0149	-0.724
MVB12B	multivesicular body subunit 12B	TC0900008776.hg.1	0.0161	-0.72
RPL9	ribosomal protein L9	TC1500010237.hg.1	0.0166	-0.718
PGAM1	phosphoglycerate mutase 1	TC1200011543.hg.1	0.017	-0.716
PRM2	protamine 2	TC1600009399.hg.1	0.0187	-0.71
SULT1A1	sulfotransferase family 1A member 1	TC1600009870.hg.1	0.0188	-0.709
CLASRP	CLK4 associating serine/arginine rich protein	TC1900008303.hg.1	0.0196	-0.706
MORF4L1	mortality factor 4 like 1	TC1500007986.hg.1	0.0202	-0.702
SPTB	spectrin beta, erythrocytic	TC1400009417.hg.1	0.0217	-0.699
NBPF20	NBPF member 20	TC0100015580.hg.1	0.0224	-0.697
SMG1	SMG1 nonsense mediated mRNA decay associated PI3K related kinase	TC1600009620.hg.1	0.0241	-0.692
GGT2P	gamma-glutamyltransferase 2, pseudogene	TC2200008078.hg.1	0.0264	-0.686
RTP2	receptor transporter protein 2	TC0300013470.hg.1	0.0294	-0.68
TRMT61B	tRNA methyltransferase 61B	TC0200012129.hg.1	0.0294	-0.679
POLR1C	RNA polymerase I and III subunit C	TC0600008095.hg.1	0.0295	-0.678
PIWIL1	piwi like RNA-mediated gene silencing 1	TC1200009399.hg.1	0.03	-0.677
PSMC1	proteasome 26S subunit, ATPase 1	TC0300007739.hg.1	0.0332	-0.671

HSPE1	heat shock protein family E (Hsp10) member 1	TC0200016584.hg.1	0.034	-0.67
MYADML2	myeloid associated differentiation marker like 2	TC1700012485.hg.1	0.0342	-0.669
H4C9	H4 clustered histone 9	TC0600014084.hg.1	0.0344	-0.668
CLDN18	claudin 18	TC0300008933.hg.1	0.0366	-0.664
PYCARD	PYD and CARD domain containing	TC1600010011.hg.1	0.0417	-0.657
H2AC25	H2A clustered histone 25	TC0100017617.hg.1	0.0417	-0.657
HYOU1	hypoxia up-regulated 1	TSUnmapped00000517.hg.1	0.0425	-0.656
OMA1	OMA1 zinc metallopeptidase	TC0100018446.hg.1	0.0427	-0.655
SLC5A10	solute carrier family 5 member 10	TC1700007145.hg.1	0.0437	-0.653
MELTF	melanotransferrin	TC0300013733.hg.1	0.044	-0.653
ZNF880	zinc finger protein 880	TC1900008696.hg.1	0.0456	-0.65
PLSCR2	phospholipid scramblase 2	TC0300014061.hg.1	0.0489	-0.646
EIF4G2	eukaryotic translation initiation factor 4 gamma 2	TC1100010092.hg.1	0.049	-0.646
NPM3	nucleophosmin/nucleoplasmin 3	TC1000011658.hg.1	0.0502	-0.644
USP40	ubiquitin specific peptidase 40	TC0200016093.hg.1	0.0502	-0.644
RAD54L	RAD54 like	TC0100008150.hg.1	0.0502	-0.643
CYP4F2	cytochrome P450 family 4 subfamily F member 2	TC1900009893.hg.1	0.0502	-0.643
CXCL1	C-X-C motif chemokine ligand 1	TC0400007840.hg.1	0.0505	-0.642
LSM5	LSM5 homolog, U6 small nuclear RNA and mRNA degradation associated	TC0700010678.hg.1	0.0502	-0.642
GLOD4	glyoxalase domain containing 4	TC1700012322.hg.1	0.054	-0.637
RPL41P2	ribosomal protein L41 pseudogene 2	TC1500006663.hg.1	0.054	-0.637
MTHFD2L	methylenetetrahydrofolate dehydrogenase (NADP+ dependent) 2 like	TC0400007848.hg.1	0.0544	-0.635
TPSG1	tryptase gamma 1	TC1600009021.hg.1	0.0544	-0.635
ZBED5	zinc finger BED-type containing 5	TC1100013144.hg.1	0.0602	-0.629
RPS6KA6	ribosomal protein S6 kinase A6	TC0X00010196.hg.1	0.0609	-0.628

UBE2A	ubiquitin conjugating enzyme E2 A	TC0X00008253.hg.1	0.0633	-0.625
TDG	thymine DNA glycosylase	TC1200008669.hg.1	0.0633	-0.625
FGF8	fibroblast growth factor 8	TC1000011657.hg.1	0.0633	-0.625
DNAJC9	DnaJ heat shock protein family (Hsp40) member C9	TC1000012564.hg.1	0.0646	-0.623
SAMM50	SAMM50 sorting and assembly machinery component	TC2200009282.hg.1	0.0648	-0.622
NCR3	natural cytotoxicity triggering receptor 3	TC0600011439.hg.1	0.0675	-0.617
PI4KB	phosphatidylinositol 4-kinase beta	TC0100015781.hg.1	0.0706	-0.614
DNAH14	dynein axonemal heavy chain 14	TC0100011755.hg.1	0.0706	-0.614
CRYBG1	crystallin beta-gamma domain containing 1	TC0600008981.hg.1	0.0726	-0.612
ZNF777	zinc finger protein 777	TC0700012995.hg.1	0.0726	-0.611
VWF	von Willebrand factor	TC1200009705.hg.1	0.0789	-0.606
EEF1E1-BLOC1S5	EEF1E1-BLOC1S5 readthrough (NMD candidate)	TC0600014237.hg.1	0.0804	-0.605
PITHD1	PITH domain containing 1	TC0100007333.hg.1	0.0804	-0.605
NUP88	nucleoporin 88	TC1700012340.hg.1	0.0804	-0.604
LINC00473	long intergenic non-protein coding RNA 473	TC0600014369.hg.1	0.0804	-0.604
GAD2	glutamate decarboxylase 2	TC1000007068.hg.1	0.0824	-0.602
ASTL	astacin like metalloendopeptidase	TC0200013510.hg.1	0.0855	-0.6
TPSD1	tryptase delta 1	TC1600006530.hg.1	0.0953	-0.593
HMHB1	histocompatibility minor HB-1	TC0500008975.hg.1	0.0953	-0.593
SLC35A2	solute carrier family 35 member A2	TC0X00009636.hg.1	0.0991	0.59
ZNF358	zinc finger protein 358	TC1900011652.hg.1	0.0964	0.592
KATNIP	katanin interacting protein	TC1600007317.hg.1	0.0953	0.593
PIAS1	protein inhibitor of activated STAT 1	TC1500007659.hg.1	0.0944	0.594
CIMIP5	ciliary microtubule inner protein 5	TC0200006714.hg.1	0.0915	0.596
MLLT1	MLLT1 super elongation complex subunit	TC1900009417.hg.1	0.0903	0.597
ESS2	ess-2 spliceosome associated protein	TC2200007973.hg.1	0.0894	0.597

SARDH	sarcosine dehydrogenase	TC0900011860.hg.1	0.0855	0.6
FOXJ1	forkhead box J1	TC1700011786.hg.1	0.0804	0.604
JPH4	junctophilin 4	TC1400010719.hg.1	0.0804	0.604
CSKMT	citrate synthase lysine methyltransferase	TC1100007865.hg.1	0.0742	0.61
INO80D	INO80 complex subunit D	TC0200015506.hg.1	0.0726	0.612
DTNA	dystrobrevin alpha	TC1800007059.hg.1	0.07	0.615
FAHD1	fumarylacetoacetate hydrolase domain containing 1	TC1600006569.hg.1	0.07	0.615
CACNB4	calcium voltage-gated channel auxiliary subunit beta 4	TC0200014620.hg.1	0.0686	0.616
LYSET	lysosomal enzyme trafficking factor	TC1400010640.hg.1	0.0691	0.616
POGZ	pogo transposable element derived with ZNF domain	TC0100015791.hg.1	0.0664	0.618
DEPDC1	DEP domain containing 1	TC0100014543.hg.1	0.0664	0.619
PPP1R27	protein phosphatase 1 regulatory subunit 27	TC1700012072.hg.1	0.0664	0.619
RABL3	RAB, member of RAS oncogene family like 3	TC0300012157.hg.1	0.0664	0.619
IQSEC2	IQ motif and Sec7 domain ArfGEF 2	TC0X00009755.hg.1	0.0663	0.62
ZNF354C	zinc finger protein 354C	TC0500009678.hg.1	0.0664	0.62
IGFBP5	insulin like growth factor binding protein 5	TC0200015678.hg.1	0.0663	0.621
EEF2KMT	eukaryotic elongation factor 2 lysine methyltransferase	TC1600009260.hg.1	0.0648	0.622
AK2	adenylate kinase 2	TC0100013641.hg.1	0.0586	0.63
SCGB1D1	secretoglobin family 1D member 1	TC1100007837.hg.1	0.0583	0.631
NR2F2	nuclear receptor subfamily 2 group F member 2	TC1500008442.hg.1	0.0571	0.632
GPAM	glycerol-3-phosphate acyltransferase, mitochondrial	TC1000011839.hg.1	0.056	0.634
TUBB4A	tubulin beta 4A class IVa	TC1900009433.hg.1	0.0542	0.636
AAK1	AP2 associated kinase 1	TC0200012961.hg.1	0.054	0.637
SEZ6L	seizure related 6 homolog like	TC2200006945.hg.1	0.0537	0.638
SLC26A2	solute carrier family 26 member 2	TC0500009076.hg.1	0.0525	0.639
TBCD	tubulin folding cofactor D	TC1700009284.hg.1	0.0525	0.639
PGPEP1	pyroglutamyl-peptidase I	TC1900007382.hg.1	0.0502	0.642

MAF	MAF bZIP transcription factor	TC1600010951.hg.1	0.0471	0.649
TP53BP2	tumor protein p53 binding protein 2	TC0100017451.hg.1	0.0447	0.652
ARHGAP1	Rho GTPase activating protein 1	TC1100010712.hg.1	0.0433	0.654
TBC1D20	TBC1 domain family member 20	TC2000008130.hg.1	0.0425	0.656
CABYR	calcium binding tyrosine phosphorylation regulated	TC1800006905.hg.1	0.0379	0.662
DNAJC14	DnaJ heat shock protein family (Hsp40) member C14	TC1200012800.hg.1	0.0379	0.663
ZNF445	zinc finger protein 445	TC0300010889.hg.1	0.0359	0.666
TNFRSF14-AS1	TNFRSF14 antisense RNA 1	TC0100012539.hg.1	0.0354	0.667
DNAJA4	DnaJ heat shock protein family (Hsp40) member A4	TC1500007967.hg.1	0.0342	0.669
UBXN1	UBX domain protein 1	TC1100011094.hg.1	0.0318	0.674
POLR1A	RNA polymerase I subunit A	TC0200016702.hg.1	0.0304	0.676
MOB3A	MOB kinase activator 3A	TC1900009204.hg.1	0.0287	0.681
SETDB2	SET domain bifurcated histone lysine methyltransferase 2	TC1300007164.hg.1	0.0287	0.681
DNAL4	dynein axonemal light chain 4	TC2200009355.hg.1	0.0284	0.682
HOXA3	homeobox A3	TC0700013529.hg.1	0.0278	0.684
RAB40B	RAB40B, member RAS oncogene family	TC1700012143.hg.1	0.0271	0.685
PBRM1	polybromo 1	TC0300011175.hg.1	0.0255	0.688
NRSN2	neurensin 2	TC2000006442.hg.1	0.0242	0.691
ZNF875	zinc finger protein 875	TC1900011721.hg.1	0.022	0.698
CD164	CD164 molecule	TC0600012814.hg.1	0.0201	0.704
VPS26C	VPS26 endosomal protein sorting factor C	TC2100008098.hg.1	0.0197	0.706
CDC42EP1	CDC42 effector protein 1	TC2200007303.hg.1	0.0196	0.706
HAPSTR2	HUWE1 associated protein modifying stress responses 2	TC0X00008586.hg.1	0.0187	0.71
ZFP64	ZFP64 zinc finger protein	TC2000009469.hg.1	0.0187	0.71
CX3CR1	C-X3-C motif chemokine receptor 1	TC0300010775.hg.1	0.0186	0.712
AAGAB	alpha and gamma adaptin binding protein	TC1500009846.hg.1	0.0166	0.718

OSBPL9	oxysterol binding protein like 9	TC0100008289.hg.1	0.0134	0.729
P2RY8	P2Y receptor family member 8	TC0X00008911.hg.1	0.0129	0.732
DMBX1	diencephalon/mesencephalon homeobox 1	TC0100008163.hg.1	0.0129	0.732
RFX5	regulatory factor X5	TC0100015783.hg.1	0.0124	0.737
GLMP	glycosylated lysosomal membrane protein	TC0100018510.hg.1	0.0109	0.745
ZNF182	zinc finger protein 182	TC0X00011363.hg.1	0.01	0.749
C11orf24	chromosome 11 open reading frame 24	TC1100011375.hg.1	0.00881	0.756
FAM107B	family with sequence similarity 107 member B	TC1000009863.hg.1	0.0077	0.763
TMT1A	thiol methyltransferase 1A	TC1200007626.hg.1	0.00743	0.765
AKT1S1	AKT1 substrate 1	TC1900012006.hg.1	0.00743	0.766
GATA6	GATA binding protein 6	TC1800006852.hg.1	0.00736	0.768
GLOD5	glyoxalase domain containing 5	TC0X00007203.hg.1	0.00736	0.769
PITX1	paired like homeodomain 1	TC0500012093.hg.1	0.00683	0.773
LIAS	lipoic acid synthetase	TC0400007285.hg.1	0.00683	0.773
COX4I2	cytochrome c oxidase subunit 4I2	TC2000007085.hg.1	0.00522	0.785
NUCB1	nucleobindin 1	TC1900008498.hg.1	0.00519	0.786
TMPPE	transmembrane protein with metallophosphoesterase domain	TC0300010664.hg.1	0.00441	0.794
TRO	trophinin	TC0X00007368.hg.1	0.00314	0.81
WBP2	WW domain binding protein 2	TC1700011773.hg.1	0.00314	0.811
DDIAS	DNA damage induced apoptosis suppressor	TC1100013075.hg.1	0.00314	0.812
OTOS	otospiralin	TC0200016309.hg.1	0.00222	0.827
WDR24	WD repeat domain 24	TC1600008972.hg.1	0.00166	0.843
ZFP36L1	ZFP36 ring finger protein like 1	TC1400009524.hg.1	0.00138	0.852
RHBDD2	rhomboid domain containing 2	TC0700008072.hg.1	0.00132	0.854
LCOR	ligand dependent nuclear receptor corepressor	TC1000008556.hg.1	0.00132	0.854
SCD5	stearoyl-CoA desaturase 5	TC0400011180.hg.1	0.00102	0.867
NKAP	NFKB activating protein	TC0X00011389.hg.1	0.000829	0.878
NMNAT2	nicotinamide nucleotide adenylyltransferase 2	TC0100016649.hg.1	0.000801	0.881

PRKAR1A	protein kinase cAMP-dependent type I regulatory subunit alpha	TC1700012296.hg.1	0.000423	0.908
MADCAM1	mucosal vascular addressin cell adhesion molecule 1	TC1900006462.hg.1	0.000137	0.95
PHC3	polyhomeotic homolog 3	TC0300013090.hg.1	0.000126	0.954
ZNF268	zinc finger protein 268	TC1200012737.hg.1	0.000039	1.009
SRRM2	serine/arginine repetitive matrix 2	TC1600006633.hg.1	0.000029	1.026
LANCL1	LanC like glutathione S-transferase 1	TC0200015608.hg.1	0.000024	1.035
ESCO2	establishment of sister chromatid cohesion N-acetyltransferase 2	TC0800007119.hg.1	0.000016	1.05
GPATCH8	G-patch domain containing 8	TC1700010831.hg.1	0.000012	1.068
ZNF780B	zinc finger protein 780B	TSUnmapped00000206.hg.1	0.000008	1.086
VCF2	VCP nuclear cofactor family member 2	TC0X00009799.hg.1	0	1.444

© 2000-2026 QIAGEN. All rights reserved.

Supplementary Table S4. Deregulated genes in *C.vulgaris* co-treated Hg^{2+}-exposed HEK-293 DMT1 cells

Symbol	Entrez Gene Name	Affymetrix Gene Code	Expr False Discovery Rate (q-value)	Expr Log Ratio
HNRNPCL1	heterogeneous nuclear ribonucleoprotein C like 1	TSUnmapped00000528.hg.1	0.000118	-0.99
VCF2	VCP nuclear cofactor family member 2	TC0X00009799.hg.1	0.000527	-0.926
MRPL28	mitochondrial ribosomal protein L28	TC1600008942.hg.1	0.000779	-0.909
TMCC1	transmembrane and coiled-coil domain family 1	TC0300012403.hg.1	0.0012	-0.889
CWC15	CWC15 spliceosome associated protein	TC1100012012.hg.1	0.00173	-0.873
EHBP1	EH domain binding protein 1	TC0200007759.hg.1	0.00208	-0.865
H2BC7	H2B clustered histone 7	TC0600007282.hg.1	0.00511	-0.827
PNMA5	PNMA family member 5	TC0X00011144.hg.1	0.0053	-0.824
H2BC13	H2B clustered histone 13	TC0600011224.hg.1	0.00572	-0.82
AMER1	APC membrane recruitment protein 1	TC0X00009877.hg.1	0.0105	-0.788
GRINA	glutamate ionotropic receptor NMDA type subunit associated protein 1	TC0800009231.hg.1	0.0108	-0.786
MARS2	methionyl-tRNA synthetase 2, mitochondrial	TC0200010374.hg.1	0.0131	-0.777
SPATA31A6 (includes others)	SPATA31 subfamily A member 6	TC0900007288.hg.1	0.0292	-0.731
TNRC6C	trinucleotide repeat containing adaptor 6C	TC1700009032.hg.1	0.0292	-0.73
ZNF573	zinc finger protein 573	TC1900010580.hg.1	0.0292	-0.73
ARHGEF26	Rho guanine nucleotide exchange factor 26	TC0300009247.hg.1	0.0313	-0.727
TADA3	transcriptional adaptor 3	TC0300010209.hg.1	0.0383	-0.714
H2AC17	H2A clustered histone 17	TC0600011235.hg.1	0.04	-0.711

NXF1	nuclear RNA export factor 1	TC1100011101.hg.1	0.0516	-0.692
ADORA2B	adenosine A2b receptor	TC1700006999.hg.1	0.0554	-0.687
NPPC	natriuretic peptide C	TC0200016047.hg.1	0.0576	-0.682
C2orf42	chromosome 2 open reading frame 42	TC0200016683.hg.1	0.0622	-0.676
FAM241B	family with sequence similarity 241 member B	TC1000007905.hg.1	0.0622	-0.675
TTC9C	tetratricopeptide repeat domain 9C	TC1100013026.hg.1	0.0622	-0.675
CDH16	cadherin 16	TC1600010602.hg.1	0.0622	-0.675
SERTAD4	SERTA domain containing 4	TSUnmapped00000211.hg.1	0.0626	-0.673
AGBL5	AGBL carboxypeptidase 5	TC0200007050.hg.1	0.086	-0.654
PCBD1	pterin-4 alpha-carbinolamine dehydratase 1	TC1000010930.hg.1	0.0946	-0.649
RAF1	Raf-1 proto-oncogene, serine/threonine kinase	TC0300010299.hg.1	0.0998	-0.644
CDC42EP1	CDC42 effector protein 1	TC2200007303.hg.1	0.0998	-0.643
RPL36A	ribosomal protein L36a	TC0X00011308.hg.1	0.0998	0.643
NAGA	alpha-N-acetylgalactosaminidase	TC2200008856.hg.1	0.0974	0.646
CNKSR1	connector enhancer of kinase suppressor of Ras 1	TC0100007444.hg.1	0.0974	0.646
SRCIN1	SRC kinase signaling inhibitor 1	TC1700010529.hg.1	0.0971	0.647
ZC3H11C	zinc finger CCCH-type containing 11C	TC0600008418.hg.1	0.0971	0.647
SENP6	SUMO specific peptidase 6	TC0600008562.hg.1	0.086	0.654
PLSCR2	phospholipid scramblase 2	TC0300014061.hg.1	0.0694	0.666
PEAK1	pseudopodium enriched atypical kinase 1	TC1500010109.hg.1	0.0694	0.666
LLPH	LLP homolog, long-term synaptic facilitation factor	TC1200012818.hg.1	0.0694	0.667
ELOA3BP (includes others)	elongin A3 family member B, pseudogene	TC1800008607.hg.1	0.0641	0.672

ZMAT2	zinc finger matrin-type 2	TC0500013238.hg.1	0.0626	0.673
ZNF574	zinc finger protein 574	TC1900008172.hg.1	0.0622	0.675
YOD1	YOD1 deubiquitinase	TC0100017118.hg.1	0.0622	0.675
CANT1	calcium activated nucleotidase 1	TC1700011923.hg.1	0.0622	0.677
PNPLA7	patatin like domain 7, lysophospholipase	TC0900012068.hg.1	0.0555	0.686
PLLP	plasmolipin	TC1600011545.hg.1	0.0555	0.686
CLDN18	claudin 18	TC0300008933.hg.1	0.0516	0.691
MIA2	MIA SH3 domain ER export factor 2	TC1400010605.hg.1	0.0516	0.691
RPL41	ribosomal protein L41	TC2200008578.hg.1	0.0471	0.699
MYH7	myosin heavy chain 7	TC1400008718.hg.1	0.0438	0.703
SCN8A	sodium voltage-gated channel alpha subunit 8	TC1200010711.hg.1	0.0429	0.705
ABHD16B	abhydrolase domain containing 16B	TC2000008094.hg.1	0.0429	0.705
HMHB1	histocompatibility minor HB-1	TC0500008975.hg.1	0.0429	0.706
CCAR1	cell division cycle and apoptosis regulator 1	TC1000007866.hg.1	0.04	0.71
CD79B	CD79b molecule	TC1700011430.hg.1	0.0376	0.716
CLIC3	chloride intracellular channel 3	TC0900012024.hg.1	0.0372	0.717
TIMP1	TIMP metallopeptidase inhibitor 1	TC0X00007149.hg.1	0.0362	0.719
TRMT61B	tRNA methyltransferase 61B	TC0200012129.hg.1	0.0292	0.73
KLK4	kallikrein related peptidase 4	TC1900011226.hg.1	0.0292	0.731
SLC49A3	solute carrier family 49 member 3	TC0400009695.hg.1	0.0284	0.736
APOBEC3A	apolipoprotein B mRNA editing enzyme catalytic subunit 3A	TC2200009269.hg.1	0.0257	0.745
IMPA1	inositol monophosphatase 1	TC0800010944.hg.1	0.0142	0.772
GNAT1	G protein subunit alpha transducin 1	TC0300007408.hg.1	0.0131	0.776
VRK1	VRK serine/threonine kinase 1	TC1400010649.hg.1	0.00923	0.797
WDR83OS	WD repeat domain 83 opposite strand	TC1900011890.hg.1	0.00844	0.802
GPR21	G protein-coupled receptor 21	TC0900008684.hg.1	0.0012	0.888

RAX2	retina and anterior neural fold homeobox 2	TC1900009308.hg.1	0.0012	0.891
PCDHB7	protocadherin beta 7	TC0500008880.hg.1	0.000278	0.95
EXOC2	exocyst complex component 2	TC0600010375.hg.1	0.0002	0.967
UQCRB	ubiquinol-cytochrome c reductase binding protein	TC0800011171.hg.1	0.000123	0.986
BMERB1	bMERB domain containing 1	TC1600011358.hg.1	0.000118	0.99
ZNF443	zinc finger protein 443	TC1900011884.hg.1	0.000118	0.992
CRYGN	crystallin gamma N	TC0700013075.hg.1	0.000118	0.998
SLC16A1	solute carrier family 16 member 1	TSUnmapped00000763.hg.1	0.000054	1.028
SMG1	SMG1 nonsense mediated mRNA decay associated PI3K related kinase	TC1600009620.hg.1	0.000011	1.082
TTYH1	tweety family member 1	TC1900008856.hg.1	0.000002	1.137
RBM24	RNA binding motif protein 24	TC0600007084.hg.1	0.000001	1.162
GGT2P	gamma-glutamyltransferase 2, pseudogene	TC2200008078.hg.1	0	1.2
TNFRSF10C	TNF receptor superfamily member 10c	TC0800007007.hg.1	0	1.42

© 2000-2026 QIAGEN. All rights reserved.
